# Targeting metacognitive change mechanisms in acute inpatients with psychotic symptoms: feasibility and acceptability of a modularized group intervention

**DOI:** 10.1007/s00406-023-01690-y

**Published:** 2023-09-23

**Authors:** Eva Gussmann, Christoph Lindner, Susanne Lucae, Peter Falkai, Frank Padberg, Samy Egli, Johannes Kopf-Beck

**Affiliations:** 1 Max Planck Institute of Psychiatry, Munich, Germany; 2 Department of Psychology, LMU Munich, Munich, Germany; 3 Department of Psychiatry and Psychotherapy, University Hospital, LMU Munich, Munich, Germany

**Keywords:** Acute inpatient setting, Acute psychosis, Mechanism-based, Metacognition, Modularized, Group therapy, Intervention

## Abstract

Emerging evidence suggests the usefulness of psychological interventions targeting metacognitive change mechanisms in patients experiencing psychosis. Although many of these patients are treated in acute psychiatric contexts, only few studies have adapted such interventions for acute inpatient settings. The present study aimed to assess the feasibility, acceptability, and preliminary clinical outcomes of a novel modularized group intervention focusing on different aspects of metacognitive change mechanisms. In particular, the intervention aims to reduce patients’ acute symptoms by enhancing cognitive insight and to relieve distress via cognitive defusion (i.e. coping). A sample of 37 participants with acute psychosis received up to nine sessions of the intervention. Baseline and post-intervention assessments were conducted for general psychopathology, psychotic symptoms, global functioning, and symptom distress. Measures of change mechanisms were assessed before and after the respective treatment module. Participants’ experiences were explored in feedback questionnaires and interviews. Recruitment, retention, and attendance rate met the pre-set feasibility benchmark of 80%. The intervention was well received by participants, who emphasised the group’s clear structure, positive atmosphere, and helpful contents. Response rates were high and linear mixed models revealed significant medium-to-large time effects on all clinical outcomes. As expected, increase in hypothesised change mechanisms cognitive insight and decrease in cognitive fusion was found. However, the uncontrolled design limits interpreting clinical effects. The study provides evidence that an intervention based on a metacognitive model is feasible and acceptable for acute inpatients with psychosis. Positive results on clinical outcomes and change mechanisms warrant further exploration in a randomized controlled trial.

**Supplementary Information:**

The online version contains supplementary material available at 10.1007/s00406-023-01690-y.

## Introduction

Psychotic spectrum disorders (PSDs), such as schizophrenia and psychotic mood disorders, affect around 3.5% of the global population [1] and are considered to be among the top 25 contributors to disability worldwide [2]. They are also among the mental illnesses associated with the highest economic costs for health care services, partially due to repeated hospitalisations [3, 4]. Internationally, as much as two-thirds of the current psychiatric inpatient population are experiencing psychosis [5], also being the group most frequently subject to involuntary admissions [6].

During acute crises, patients with PSDs can pose high risks to themselves and others, requiring treatment in acute psychiatric inpatient wards (also known as secure, locked or acute wards) [7]. In contrast to open wards, where inpatients are treated after their most severe symptoms have subsided, acute psychiatric inpatient wards often focus primarily on psychopharmacological treatment rather than psychological interventions, resulting in on-going patient dissatisfaction [8]. The lack of psychotherapeutic activity moreover contrasts with treatment guidelines, which recommend psychological interventions for psychosis already in the acute treatment stage [9, 10] to improve patients' functioning and support recovery [11, 12]. Interestingly, recent systematic reviews and meta-analyses report heterogeneous findings for guideline-recommended cognitive behavioural therapy for psychosis (CBTp) in acute psychiatric inpatient settings [13–15]. However, promising evidence supports the efficacy of third-wave therapies like Acceptance and Commitment Therapy (ACT) and CBT approaches integrating third-wave components, such as Metacognitive Training (MCT) [13–15].

Disorder-specific CBTp protocols aim to change the appearance and nature of psychotic symptoms [16]. In contrast, third-wave therapies focus on how individuals process and manage experiences while encouraging a mindful and accepting attitude towards them [17]. They also often directly focus on targeting transdiagnostic change mechanisms that are thought to positively impact treatment outcomes [18]. In this context, change mechanisms rely on psychological processes found to be responsible for the onset and maintenance of disorders [17]. In the case of psychosis, third-wave approaches have a particular interest in various aspects of impaired metacognitive processes and associated metacognitive change mechanisms [19, 20]. More precisely, approaches try to enhance patients’ critical awareness of own thoughts (“thinking about thinking”) [21] in order to change immediate thought-related reactions [19]. MCT, for example, aims to promote patients’ cognitive insight via raising metacognitive awareness and knowledge for cognitive biases [22] and has demonstrated significant effectiveness in reducing positive symptoms [16, 23, 24]. ACT on the contrary, although not categorized specifically as a metacognitive therapy, also incorporates several metacognitive elements. Key ACT concepts such as mindfulness, acceptance, cognitive defusion (ACT term for cognitive distancing), and value commitment [25], are associated with metacognitive awareness and functional metacognitive goals and strategies [26–28]. With regard to acute inpatients with PSDs, ACT-based interventions have been shown to reduce general psychopathology and rehospitalisation rates [11, 29, 30].

While altering cognitive responses to experiences instead of directly challenging them seems to be especially helpful in treating acute psychotic symptoms [31], existing evidence has to be approached with caution [13–15]. Apart from the current small evidence base and methodological shortcomings, most of the metacognitive interventions for psychosis that have been studied were originally developed for outpatients [32–35] or for inpatients with mild to moderate symptoms [22] and were not tailored to fit the unique characteristics of acute psychiatric settings and inpatients [13, 36]. These include restrictive environments, high economic pressure, brief admissions, and acutely unwell patients likely to pose high risks, have multiple disorders, cognitive difficulties and low motivation for treatment [7]. Given the urgent need to improve acute inpatient care, yet a remaining substantial research gap, studies are needed to investigate the feasibility and effectiveness of adapted interventions [37].

Therefore, the present research aimed to examine the feasibility and acceptability of a novel modularized and mechanism-based treatment, while evaluating preliminary clinical outcomes and alterations in potential change mechanisms. More precisely, the current study extended our previous work [36] on designing an adapted metacognitive treatment using Intervention Mapping [38] as suggested by best practice guidelines on complex intervention development [39]. Specifically, the novelty of the intervention (see Supplementary Material and our previous work for details) [36] is that it (1) focuses directly on underlying transdiagnostic metacognitive change mechanisms (cognitive insight and cognitive defusion) rather than on specific symptom content, thus following a current paradigm shift towards mechanism-based psychotherapeutic treatments [18, 40, 41], (2) combines and integrates different existing evidence-based therapeutic approaches in a hybrid and modularized approach allowing for tailored treatments and greater flexibility [42, 43], (3) is delivered in a group format to take advantage of social support and optimal resource use [44, 45], and (4) adapts all therapeutic elements to be brief, flexible and low-key to meet the needs of acute inpatients with PSDs [37].

We hypothesised that (1) feasibility and acceptability measures would exceed the 80% benchmark necessary to proceed to a fully powered effectiveness randomized controlled trial (RCT) [46]. Furthermore, we assumed that (2) participants would show significant improvements (compared to baseline) on general psychopathology, positive and negative symptoms, symptom distress, symptom severity, and functioning, and that (3) targeting metacognitive treatment mechanisms would lead to positive changes, as evidenced by increased cognitive insight and decrease in cognitive fusion (i.e. greater cognitive defusion from internal experiences).

## Materials and methods

### Procedure and participants

**Fig. 1:**
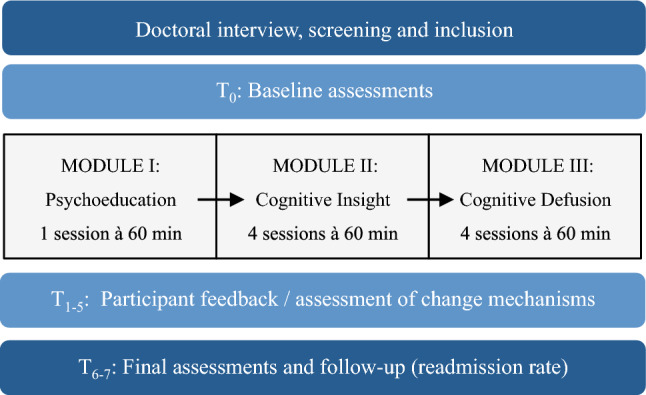
The study and intervention design

Between May 2021 and February 2022, we recruited a total of *N* = 37 participants from the acute psychiatric inpatient ward of the Max Planck Institute of Psychiatry in Munich, Germany for the study. Within this period, nine group therapy cycles were conducted. After a standardized screening process, eligible participants were briefed about the study’s procedures and written informed consent was obtained. Enrolment into the group therapy was possible at the beginning of each module. The screening procedure and all rater-based assessments were either conducted by a clinical psychologist or psychiatrist in training. Inclusion criteria were: (1) aged between 18 and 70; (2) diagnosed with a PSD (ICD-10 codes F20-39); and (3) able to give informed consent. Exclusion criteria were: (1) severe neurological or internal concomitant diseases; (2) IQ < 80, severe learning disability, brain damage or pervasive developmental disorder; and (3) missing eligibility for psychotherapy because of missing language skills, hostile or uncooperative behaviour. Our sample size of *N* = 37 participants exceeded the suggested benchmark of *N* = 20 participants required to evaluate the feasibility, acceptability and preliminary effectiveness of a group therapy intervention [47], also for studies with PSDs [48–50]. Following guidelines on conducting feasibility studies, we employed a non-randomised exploratory pre-post design closest to a Phase II early clinical trial [51, 52] (see Fig. 1), suitable for assessing and maximizing the intervention’s potential effectiveness for future research [39]. Outcome measures were taken at baseline (timepoint T_0_), before and after each therapy module (timepoints T_1_, T_2_, T_3_, T_4_, T_5_) and post intervention (timepoint T_6_). Rehospitalisation data was examined up to 12 months after completion (timepoint T_7_). Our study received approval from the ethics committee of the Medical Faculty at Ludwig Maximilian University Munich (PNO-21-0025) and was pre-registered in ClinicalTrails.gov (TRN04874974-2021.04.26).

### Modularized metacognitive group intervention

We designed the metacognition-focused and modularized group therapy as an experimental group in addition to the already existing mechanism-based therapy concept of the acute psychiatric inpatient ward (see Supplementary Methods 1 and Supplementary Fig. 1 for an overview) [36]. The five-week group intervention consisted of nine stand-alone sessions (two per week) divided into three modules targeting various metacognitive and social change mechanisms, with the overall goal of enhancing cognitive flexibility (see Supplementary Fig. 2 for underlying therapy model). Modules I and II aimed to enhance attentiveness to internal experiences by promoting metacognitive awareness and knowledge and hence cognitive insight for cognitive distortions. Module III focused on reducing distress and automatic relational responses through cognitive defusion and therewith strengthen metacognitive goals and strategies. Module I contains mainly psychoeduactional material and exercises on metacognition (cognitive biases and dysfunctional coping strategies), adapted in a transdiagnostic way from the Metacognitive Training for depression [53]. Therapy contents for Module II were adapted from the “acute version” of the Metacognitive Training for psychosis by Moritz and Woodward [22, 54]. Module III includes adapted exercises from the Metacognitive Therapy by Wells and Matthews [35] and the Acceptance and Commitment Therapy by Hayes [34]. A description of sessions’ contents can be found in Supplementary Table 1. To address the specific characteristics of acute inpatients with psychotic symptoms, such as low illness insight, treatment resistance [55], severe cognitive deficits [56], and comorbid diagnoses [57], we designed the contents to be transdiagnostic, experiential, and easy-to-comprehend. Information was presented on simple PowerPoint slides, group sizes were kept small with no more than seven participants, and each session lasted a maximum of 60 min. Sessions were carried out by a psychotherapist trained in CBT who adopted an empowering and self-disclosing therapeutic attitude [58]. Due to the naturalistic study design, participants were allowed to participate in one other group therapy, received weekly individual psychotherapy sessions and additional routine care (described in Supplementary Methods 2) within the acute inpatient setting. Any other interventions participants were involved in were documented. Risk assessments and evaluations were conducted regularly during group sessions and team meetings with medical staff. Pre-specified adverse events included: symptom aggravation, new symptoms, treatment misuse, increased suicidality, and negative impact on work or social network. The assessments were documented using standardized checklists proposed by Linden [59]. In case of a serious adverse event (attempted suicide) related to the intervention, the termination of the study was determined.

### Outcome measures

Primary outcomes consisted of measures operationalized to assess the feasibility and acceptability of the intervention and study evaluation design. Secondary outcomes included multiple clinical measures that were used to evaluate the preliminary effectiveness of the intervention. Demographic information was collected at baseline via a self-reported questionnaire, supplemented by the clinical record. Baseline medication and any changes during the course of the study were recorded using participant’s medical records. Table 1 presents an overview of all study instruments and the sequence of their administration at each of the timepoints.

**Table 1:** Measurements across each timepoint

Time point	Baseline	Intervention					Post-intervention	Follow-up
	T_0_	T_1_	T_2_	T_3_	T_4_	T_5_	T_6_	T_7_
WEEK	0	1	2	3	4	5	5	12 months
Demographics	X							
Treatment regime	X						X	
Primary outcome measures								
General feasibility measures	X	X	X	X	X	X	X	
Participant feedback questionnaire		X		X		X	X	
Semi-structured interview							X	
Secondary outcome measures								
Positive and Negative Syndrome Scale	X						X	
Psychotic Symptom Rating Scale	X						X	
Global Assessment of Functioning	X						X	
Clinical Global Impression Scale	X						X	
World Health Organization Disability Assessment Schedule	X						X	
Beck Cognitive Insight Scale			X	X				
Cognitive Fusion Questionnaire					X	X		
Readmission rate								X

*Note.* General feasibility measures included: eligibility rate, consent rate, trial entry rate, completion and missing data rate, retention rate, dropout rate, attendance rate and adverse events. Participant feedback questionnaires were handed out after each module and rated the participants’ subjective satisfaction with the corresponding module. Insights from therapy and suggestions for  improvement were interrogated from selected participants in semi-structured interviews after completing the whole intervention

#### Primary outcome measures

Using the CONSORT extension to pilot and feasibility studies [60] feasibility data included: (1) eligibility rate, (2) consent rate, (3) trial entry rate, (4) completion and missing data rate, (5) retention rate, (6) dropout rate, (7) patient engagement, and (8) adverse events. Acceptability, subjective effectiveness and participants’ treatment satisfaction with each module and the whole intervention was measured with a five-point Likert scale self-report questionnaire (see Supplementary Methods 3) adapted from Moritz and Woodward [61]. Additionally, all participants were invited to give general feedback on the group therapy and study conditions in semi-structured interviews conducted at study completion (see Supplementary Methods 4). Following guidelines on evaluating pilot studies [46, 62], feasibility and acceptability criteria were benchmarked a priori with a traffic light system on recruitment, retention and attendance rate as well as patients’ overall treatment satisfaction: red (not feasible < 60%), yellow (modify intervention and protocol ≥ 60% < 80%), and green (continue without modifications > 80%) [63–65].

#### Secondary clinical outcome measures

General psychopathology as well as negative and positive symptoms were rated with the Positive and Negative Syndrome Scale (PANSS), a clinician-administered 30-item semi-structured interview [66]. On the three different scales (positive, negative and global symptom scale), items are scored on a seven-point Likert scale between 1 (not present) and 7 (severe). The PANSS demonstrates strong internal consistency, indicated by a Cronbach's α = 0.73 and a high inter-rater reliability (between 0.83 and 0.87) [67].

Symptom distress was measured with the Psychotic Symptom Rating Scale (PSYRATS), a 17-item clinician-administered semi-structured interview. On two different subscales (auditory hallucinations and delusions), different dimensions (e.g. controllability, severity and intensity of distress and disruption) of hallucinations and delusions are rated between 0 (not present) and 4 (highest possible distress). The PSYRATS is reported to have a good internal consistency with a high inter-rater reliability (between 0.79 and 1.00) [68].

The level of functioning was assessed using the Global Assessment of Functioning (GAF), a clinician-administered rating scale. The GAF scale considers both symptoms and functionality, and its scores range from 1 (indicating a risk of self-harm or harm to others) to 100 (suggesting the absence or minimal presence of symptoms). It demonstrates a good internal consistency with Cronbach’s α = 0.70 [69, 70], but has been criticised for its weak inter-rater reliability [71].

Symptom severity and treatment response to the intervention was rated on the Clinical Global Impression (CGI) rating scales, a one-item clinician-administered assessment [72]. On the severity scale (CGI-S), the severity of an individual’s illness is evaluated relative to the clinician’s past experience on a seven-point Likert scale from 1 (not at all ill) to 7 (among the most extremely ill patients). The improvement scale (CGI-I) quantifies the individual’s improvement or worsening since the start of the intervention from 1 (very much improved) to 7 (very much worse) [73]. The CGI is one of the most widely used rating scales in mental health trials and several studies demonstrated its validity by linkage to rating scales such as the PANSS [74].

Disability and functional impairment were estimated using the World Health Organization Disability Assessment Schedule 2.0 (WHODAS-2.0), a 12-item self-report questionnaire [75]. The six disability dimensions (social, cognitive, society, self-care, household, and mobility) of the International Classification of Functioning (ICF) [76] serve as subscales in the questionnaire. These are rated using a five-point Likert scale (1 = no disability to 5 = very strong disability). The WHODAS shows good reliability (Cronbach’s α = 0.89) [77, 78]. As suggested in the literature, inpatients with psychosis tend to overestimate their functioning [79], so we introduced an additional rater-corrected WHODAS score when a participant lacked the insight to answer the questions objectively. Following the approach of Gspandl et al. [80] and the DSM-5’s WHODAS-2.0 Clinician Administration guide [81], we used information from proxy respondents such as family members and carers, as well as clinical judgement, to record a question-by-question “corrected” score alongside the participant's self-reported “raw” score.

The hypothesised metacognitive change mechanism of cognitive insight was determined using Beck’s Cognitive Insight Scale (BCIS), a 15-item self-report questionnaire. The BCIS contains two subscales, self-reflection and self-certainty regarding one’s thoughts and experiences, which are rated using a four-point Likert scale from 0 (do not at all agree) to 3 (agree completely). It presents acceptable internal consistency with Cronbach's α = 0.60–0.68 [82].

To assess the potential change mechanism of cognitive defusion, the Cognitive Fusion Questionnaire (CFQ) was used. The seven-item self-report questionnaire measures the extent to which an individual's behaviour is influenced by thoughts (cognitive fusion), using a seven-point Likert scale ranging from 1 (never true) to 7 (always true). Previous studies have demonstrated its high internal consistency (Cronbach’s α = 0.89–0.93) [83, 84].

Rehospitalisation rates (to the same unit or psychiatric hospital) during the follow-up period were monitored exploratory using internal patient chart records.

### Data analysis

In line with the CONSORT guidelines on reporting pilot and feasibility studies [60], we focused the analysis on descriptive statistics for feasibility and acceptability measures using frequencies and percentages. Thematic analysis [85], a systematic approach to organize, encode, and analyse patterns (themes) within qualitative data, was employed for the semi-structured interviews. Changes in dosages of psychotropic medication from baseline to post-intervention were compared by computing dose equivalents [86] and conducting parametric (paired t-tests) or non-parametric (Wilcoxon’s signed ranks) tests depending on the data’s distribution.

Intraclass Correlation Coefficients (ICC) for all secondary outcomes (0.25–0.67) provided evidence for a nested data structure [87, 88], so we used linear mixed models (LMMs, for details see e.g. [89]) via the maximum likelihood method to estimate participants’ changes on secondary clinical measures (i.e., post–pre treatment comparison) [90]. In all our LMMs, the measurement occasions of the outcomes were represented as a binary-coded time variable with 0 (i.e., baseline measure before treatment) and 1 (i.e., post-intervention measure). The time variable was added as a fixed effect on the within-participant level, while participants’ ID was treated as a random effect [91, 92]. All our LMMs controlled for potential confounders by including the covariates sex, age, psychotherapeutic treatment dosage (group and total), and medication change scores (antipsychotic and antidepressant), that we selected based on previous research findings [93].

For investigating clinically significant changes over treatment time, we referred to the recommended criteria of 25% and 50% of improvement indicated by percentage of PANSS total scores reduction from baseline and to the CGI-improvement scale cut-offs [94, 95]. Finally, for exploratory rehospitalisation rates, we calculated the proportion of participants readmitted to the same unit or hospital within the follow-up period. All statistical analyses were conducted using R Software, version 4.1.2 [96].

## Results

Baseline demographic and clinical characteristics and changes in the participants’ medication regime are shown in Tables 2 and 3. There were no significant differences in the antipsychotic medication dosages between baseline and post-intervention. However, we found significant changes for antidepressants and benzodiazepines.

**Table 2:** Demographic and clinical characteristics of participants (*N* = 37)

Baseline characteristic	F_*N*_ (%); *M* (SD)
Sex	
Male	16 (43.24%)
Female	21 (57.75%)
Age (years)	45.43 (15.09)
Ethnicity	
Caucasian	32 (86.49%)
Hispanic	0 (0%)
African German	2 (5.41%)
Asian German	3 (8.11%)
Family Status	
Single	16 (43.24%)
Partnership/Married	15 (40.54%)
Separated/Divorced/Widowed	6 (16.22%)
With children	17 (45.94%)
Years of education	
Low (≤ 10 years)	16 (34.24%)
Middle (≥ 12 years)	15 (40.54%)
High (≥ 15 years)	6 (16.22%)
Occupation	
Unemployed	16 (43.22%)
In retirement	7 (18.92%)
Student	4 (10.81%)
Employed	10 (27.03%)
Primary diagnosis	
F20-29 (Psychosis-spectrum disorders)	29 (78.38%)
F30-39 (Psychotic mood disorders)	8 (21.62%)
Psychotic symptoms (self-report)	
Delusions only	15 (40.54%)
Hallucinations only	2 (5.41%)
Delusions + Hallucinations	20 (54.05%)
Duration of illness (psychosis) in years	7.39 (9.29)
Refractory status	12 (32.43%)
Number of comorbid psychiatric diagnoses	
0	21 (56.76%)
1	9 (24.32%)
2	4 (10.81%)
3	3 (8.11%)
Number of previous hospitalisations	5.54 (4.59)
Type of hospital admission	
Involuntary	7 (18.91%)
Voluntary	30 (81.08%)
Previous psychotherapeutic experience	
None	4 (10.81%)
Received (In- and/or outpatient)	33 (89.19%)
Therapy motivation (self-report from 0 to 100%)	83.92 (24.58)

*Note.* Refractory status was assessed using Kane’s criteria on treatment-resistant schizophrenia [123]. Comorbid diagnoses included ICD diagnoses from F06 (n = 2), F10 (n = 2), F12 (n = 5), F13 (n = 2), F17 (n = 3), F19 (n = 1), F32 (n = 1), F42 (n = 2), F44 (n = 1), F45 (n = 1), F60 (n = 1), F84 (n = 2), F90 (n = 2) and Z73 (n = 1)

**Table 3:** Participants’ medication regime at baseline and post-intervention

Type, number and mean dose equivalent	Baseline	Post-intervention	*t*	*V*	*p*
	*n* (%)	*n* (%)			
Antipsychotics					
0	1 (2.70%)	2 (5.41%)			
1	21 (56.76%)	17 (45.94%)			
2	6 (16.22%)	11 (29.73%)			
≥ 3	9 (24.32%)	7 (18.92%)			
Mean dose equivalent in mg^a^ (SD)	14.26 (11.75)	16.02 (7.83)	0.99		0.163
Antidepressants					
0	23 (62.16%)	20 (54.05%)			
1	12 (32.43%)	12 (32.43%)			
≥ 2	2 (5.41%)	5 (13.51%)			
Mean dose equivalent in mg^b^ (SD)	9.99 (14.98)	17.90 (23.23)		108	0.003
Mood stabilizers					
0	34 (91.91%)	36 (97.30%)			
1	3 (8.11%)	1 (2.70%)			
Benzodiazepines					
0	21 (56.76%)	25 (67.57%)			
1	16 (43.24%)	12 (32.43%)			
Mean dose equivalent in mg^c^ (SD)	1.12 (1.71)	0.43 (0.82)		16.5	0.004

*Note.* Table format adapted from Boege et al. [63]

For normally distributed data, parametric tests were used. For skewed distributions non-parametric Wilcoxon tests were used

^a^Dosages converted to Olanzapine equivalent

^b^Dosages converted to Fluoxetine equivalent

^c^Dosages converted to Lorazepam equivalent

### Feasibility and acceptability

**Fig. 2:**
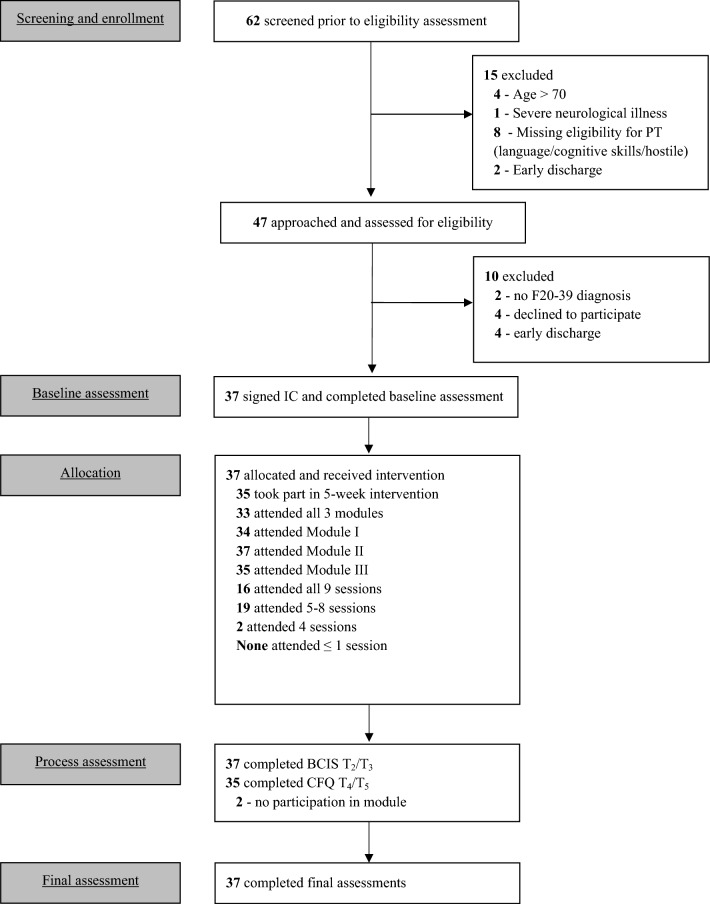
CONSORT flow diagram of the recruitment, assessment and treatment process. Feasibility measures were defined as: 1) eligibility rate (proportion of those eligible to participate as a percentage of those screened); 2) consent rate (proportion of those who signed the informed consent as a percentage of those who were approached to participate); 3) trial entry rate (proportion of those who consented and completed baseline measures); 4) completion and missing data rate (proportion of assessments completed at each time point including screening, baseline, intervention and final meeting and reasons for missing data); 5) retention rate (proportion of those who began the treatment and completed all three modules); 6) dropout rate (patients you entered the trial, attended at least one therapy session and dropped out before completing at least one module); 7) patient engagement (proportion of those attending at least two thirds of the intervention, i.e. six sessions, as well as the reasons for non-attendance); and 8) adverse events (any unwanted events related to the intervention)

The study’s CONSORT chart is illustrated in Fig. 2. In terms of feasibility, the eligibility and consent rates were 75.8% and 78.7% respectively, while the trial entry rate was 100%. The completion rate for all clinical assessments and between-module feedback questionnaires was high at 99.4%. All participants attended at least one module, resulting in a dropout rate of 0%. 33 of the 37 participants completed all three modules leading to an overall retention rate of 89.2%. Session attendance was consistently high with 86.5% of participants attending at least six sessions, i.e. two thirds of the total intervention. Five participants experienced a total of seven adverse events over the course of the study. These included one negative impact on work, one appearance of new symptoms and five symptom deteriorations. None was related to the intervention.

Participants’ acceptability and satisfaction with the group intervention was high (see Table 4), with 85.2%, 91.9%, 91.4% and 80% of the participants rating their treatment satisfaction for Modules I, II, III, and the overall treatment respectively with the highest possible rating (applies to a great extent or applies exactly). Illustrative open-ended feedback quotes (see Table 4) on each module and on the group therapy as a whole further support participants' satisfaction with and positive insights gained from the group therapy. Greater details concerning attendance data, complete presentation of the qualitative feedback on the questionnaires, participation in supplementary treatments and therapy content of individual therapies can be found in Supplementary Tables 2–5.

**Table 4:** Participants’ quantitative and qualitative feedback on each module and the overall treatment

Numeric items 1–12	Module Psychoeducation (*n* = 34)	Module Cognitive Insight (*n* = 37)	Module Cognitive Defusion (*n* = 35)	Overall treatment (*n* = 35)
	*M* (SD) [%] Positive appraisal^a^	*M* (SD) [%] Positive appraisal^a^	*M* (SD) [%] Positive appraisal^a^	*M* (SD) [%] Positive appraisal^a^
1. Useful and helpful	3.26 (0.90) [82.4]	3.35 (0.92) [83.78]	3.23 (0.97) [82.86]	3.37 (1.03) [88.57]
2. Understandable contents	3.35 (0.81) [85.29]	3.54 (0.73) [91.89]	3.39 (0.83) [80.00]	3.34 (0.91) [85.71]
3. Applicable in everyday life	2.74 (1.02) [64.71]	2.94 (1.17) [65.57]	3.71 (0.94) [57.14]	2.80 (1.13) [60.00]
4. Coping ideas	2.44 (1.21) [61.76]	2.84 (1.19) [59.46]	3.85 (1.21) [68.57]	2.74 (1.34) [60.00]
5. Clear rationale	3.03 (0.94) [76.47]	3.35 (0.92) [83.78]	3.26 (1.16) [82.86]	3.34 (1.00) [82.86]
6. Fun sessions	2.62 (1.18) [64.71]	2.84 (1.21) [64.86]	2.88 (1.02) [68.57]	2.89 (1.11) [68.57]
7. Boring sessions	1.38 (1.3) [23.53]	1.32 (1.31) [21.62]	1.36 (1.32) [22.86]	1.29 (1.38) [20.00]
8. Group format	3.15 (0.97) [76.47]	3.32 (1.06) [83.78]	3.47 (0.61) [91.43]	3.26 (1.04) [85.71]
9. Comfort in group	3.15 (1.08) [76.47]	3.24 (1.01) [78.38]	3.18 (0.97) [80.00]	3.09 (1.09) [80.00]
10. Important for treatment	2.68 (1.15) [61.76]	2.76 (1.23) [67.57]	2.91 (0.97) [65.71]	2.74 (1.20) [60.00]
11. Recommendation for others	3.12 (0.95) [79.41]	3.35 (0.98) [83.78]	3.53 (0.99) [82.86]	3.26 (1.22) [85.71]
12. Overall satisfaction	3.26 (0.79) [85.29]	3.46 (0.84) [91.89]	3.43 (1.01) [91.43]	3.31 (1.11) [80.00]

Categorical items 13–14	N (missing n)	N (missing n)	N (missing n)	N (missing n)
13. Number of sessions	34 (2)	37 (1)	35 (2)	35 (1)
	n (%)	n (%)	n (%)	n (%)
Too few	1 (3.15%)	5 (13.88%)	3 (9.09%)	1 (2.94%)
Just right	29 (90.62%)	29 (80.55%)	25 (75.76%)	28 (82.35%)
Too many	2 (6.25%)	2 (5.55%)	5 (15.15%)	5 (14.71%)
	N (missing n)	N (missing n)	N (missing n)	N (missing n)
14. Duration of sessions	34 (0)	37 (1)	35 (0)	35 (0)
	n (%)	n (%)	n (%)	n (%)
Too short	2 (5.88%)	3 (8.33%)	3 (8.57%)	2 (5.71%)
Just right	27 (79.41%)	27 (75.00%)	25 (71.43%)	26 (74.29%)
Too long	5 (14.70%)	6 (16.66%)	7 (20.00%)	7 (20.00%)

Open feedback items 15–16	Example quotes Module Psychoeducation	Example quotes Module Cognitive Insight	Example quotes Module Cognitive Defusion	Example quotes overall treatment
15. Insights from the module	“This gave me motivation to fight.” (P56) “I can change something about the way I think and therewith, I can change my problems.” (P80)	“Strengthened self-worth.” (P70) “Careful with JTC, wait until you know what the other wants.” (P53) “Not to cling to thoughts and go into the thought trap.” (P77)	“One should get help when having problems.” (P90) “Notice my thoughts actively and distinguish whether they are helpful or not and how much they influence my behaviour.” (P33) “I don’t have to control my thoughts, thoughts are thoughts and not facts.” (P58)	“The group helped me to see that many fight against the same problems and that there are many ways to cope with them.” (P22) “Taking metacognitive perspective, balancing thoughts, not taking decisions with too few information.” (P47)
16. Ideas for improvement	“Thoughts versus voices.” (P80) “Skills and how to stop thoughts.” (P20)	“How to handle incomplete information, decision aids for accepting things.” (P24) “I need more tips on how to train my memory. I know this doesn't fit with the problems of the others.” (P16)	“Some things were too fast.” (P58) “Discuss thoughts during acute psychosis.” (P49)	“Personal topics and examples.” (P53) “Talking about topics in individual session to recognize what helps me.” (P20)

*Note.* The feedback questionnaires use a five-point Likert scale ranging from 0 (does not apply at all) to 4 (applies exactly) for items 1–12. For items 13–14, participants decide between three levels of a rating scale. Item 15 and 16 are open-ended questions. Example quotes are represented in this table and marked with the corresponding participant number. A more detailed table with all quotes can be found in the Supplementary Material (Supplementary Table 3)

^a^Positive appraisal: The pooled relative number of participants that answered the item with 3 (applies to a large extent) and 4 (applies exactly)

25 of the 37 participants agreed to participate in the voluntary semi-structured feedback interview following study completion. Regarding positive group aspects, topics included helpful therapy contents, e.g. defusion techniques, and supporting environment, e.g. positive group atmosphere (see Supplementary Fig. 3). Themes identified for insights through therapy were gains in metacognitive abilities, e.g. thought awareness and recontextualisation (see Supplementary Fig. 4). Themes related to intervention deficiencies included e.g. tight session schedules and too few practical exercises (see Supplementary Fig. 5). Lastly, themes concerning the study and group setup comprised e.g. shortening session duration (see Supplementary Fig. 6). Examples of participants’ quotes and identified codes that support themes can be found in Supplementary Table 6 and 7.

### Secondary clinical outcomes

The results of the LMMs (see Table 5) revealed significant medium-to-large post-intervention reductions from baseline for all secondary clinical outcomes, except for the self-rated WHODAS measuring disabilities and functional impairments. More precisely, we found reduced general psychopathology (*b* = − 17.03, 95% CI: − 23.78, − 10.27, *d* = -0.93), positive (*b* = − 6.59, 95% CI: − 8.64, − 4.53, *d* = − 1.24) and negative symptoms (*b* = -3.05, 95% CI: − 5.02, − 1.08, *d* = − 0.53), symptom distress (*b* = -12.07, 95% CI: − 16.88, − 7.26, *d* = − 0.99), symptom severity (*b* = − 1.04, 95% CI: − 1.56, − 0.53, *d* = − 0.97) and increased levels of global functioning (*b* = 19.72, 95% CI: 14.89, 24.56, *d* = 1.58). We also found a post-treatment reduction for the adjusted WHODAS-score (*b* = − 5.26, 95% CI: − 7.94; − 2.57, *d* = − 0.67). Regarding hypothesised change mechanisms, we found a significant post-module reduction in self-certainty after Module II (*b* = − 1.64, 95% CI: − 2.84, − 0.45, *d* = − 0.45) and in cognitive fusion after Module III (*b* = − 4.52, 95% CI: − 8.24, − 0.81, *d* = − 0.43). Time effects on secondary clinical outcomes were not alternatively explained by differences in sex, age, psychotherapeutic treatment dosage, or medication change since we controlled for these covariates in our LMMs.

**Table 5:** Effect of time on secondary outcome measures using linear mixed models

Secondary outcome measures	Min–Max	Baseline M (SD)	Post-intervention M (SD)	Time Coefficient^a^ *b*	*SE*	95% CI	*p*	Cohen’s *d*^*b*^
PANSS total score	30 to 210	82.32 (18.81)	62.24 (17.92)	− 17.03	12.00	[− 23.78, − 10.27]	< 0.001	− 0.93
PANSS-positive scale	7 to 49	20.35 (5.89)	13.73 (4.68)	− 6.59	3.61	[− 8.64, − 4.53]	< 0.001	− 1.24
PANSS-negative scale	7 to 49	20.08 (6.09)	15.84 (5.50)	− 3.05	3.73	[− 5.02, − 1.08]	0.008	− 0.53
PANSS-global scale	16 to 112	41.89 (10.03)	32.68 (9.45)	− 7.39	7.86	[− 11.68, − 3.09]	0.004	− 0.76
PSYRATS total score	0 to 68	22.62 (13.46)	10.94 (10.72)	− 12.07	8.29	[− 16.88, − 7.26]	< 0.001	− 0.99
PSYRATS-Delusions scale	0 to 24	14.48 (4.75)	7.89 (6.00)	− 5.84	3.19	[− 7.68, − 3.99]	< 0.001	− 1.08
PSYRATS-Auditory hallucinations scale	0 to 44	8.13 (13.18)	3.05 (7.78)	− 6.23	7.78	[− 10.63, − 1.83]	0.014	− 0.58
GAF	1 to 100	34.94 (12.55)	56.19 (12.40)	19.72	8.54	[14.89, 24.56]	< 0.001	1.58
CGI-severity scale	1 to 7	5.73 (0.83)	4.59 (1.26)	− 1.04	0.98	[− 1.56, − 0.53]	0.001	− 0.97
WHODAS total score	12 to 60	32.54 (10.40)	28.28 (8.66)	− 1.91	5.23	[− 5.09, 1.28]	0.279	− 0.20
WHODAS-cognitive scale	2 to 10	5.70 (2.39)	4.89 (2.21)	− 0.42	1.41	[− 1.26, 0.41]	0.359	− 0.18
WHODAS-society scale	2 to 10	6.76 (2.28)	6.08 (2.19)	0.00	1.57	[− 0.90, 0.91]	0.996	0.00
WHODAS-social scale	2 to 10	5.59 (2.58)	4.76 (1.88)	− 0.58	2.10	[− 1.73, 0.57]	0.359	− 0.26
WHODAS total score-rater-adjusted	12 to 60	36.67 (8.20)	29.38 (7.57)	− 5.26	4.54	[− 7.94, − 2.57]	0.001	− 0.67
Potential change mechanisms		Pre-module	Post-module					
BCIS composite score	− 18 to 27	3.76 (7.21)	4.13 (5.44)	0.73	3.58	[− 1.42, 2.89]	0.536	0.11
BCIS-self-reflectiveness	0 to 27	12.70 (5.04)	12.16 (3.71)	− 0.91	2.95	[− 2.63, 0.81]	0.339	− 0.21
BCIS-self-certainty	0 to 18	8.94 (3.99)	8.03 (3.24)	− 1.64	1.99	[− 2.84, − 0.45]	0.017	− 0.45
CFQ	7 to 49	27.86 (10.69)	24.31 (10.34)	− 4.52	6.24	[− 8.24, − 0.81]	0.033	− 0.43

*Note.* BCIS: Beck Cognitive Insight Scale; CGI: Clinical Global Impression; CI: Confidence interval; CFQ: Cognitive Fusion Questionnaire; GAF: Global Assessment of Functioning; SE: Standard error of random effects; PANSS: Positive and Negative Syndrome Scale; PRSYRATS: Psychotic Symptom rating scale; WHODAS: World Health Organization Disability Assessment Schedule. The BCIS was measured before and after Module II (Cognitive Insight), the CFQ was assessed before and after Module III (Cognitive Defusion). All other measures were taken at baseline and after completing the whole intervention. For the WHODAS scores, a rater-adjustment was introduced as participants partly overestimated their functioning [80]

^a^Adjusted time coefficient representing mean differences between post-intervention scores and baseline scores. All LMMs controlled for the covariates sex, age, psychotherapeutic treatment dosage (group and total) and medication changes in antidepressants and antipsychotics, included as random effects in the LMMs

^b^Adjusted effect sizes were calculated as the square root of the adjusted post-baseline mean difference divided by the pooled standard deviation estimates

Analyses of clinically significant change in means of relative changes in PANSS total scores from baseline are shown in Table 6 [94]. At post-intervention, 75% of the refractory and 36% of the non-refractory participants fulfilled the response criteria. According to responder cut-off definitions on the CGI-improvement scale (at least minimally better) [94], 91.9% of the participants responded to the treatment. At 12-month follow-up, 16.2% of the participants were readmitted to our hospital one or more times (up to three times).

**Table 6:** Percentage changes from baseline in PANSS total scores as responder rates

	< 0 reduction (i.e. increase) *n (%)*	0–24% PANSS reduction *n (%)*	25–49% PANSS reduction *n (%)*	50–74% PANSS reduction *n (%)*	75–100% PANSS reduction *n (%)*
Refractory participants (N = 12)	0 (0)	2 (16.7)	7 (58.3)	2 (16.7)	1 (8.3)
Non-refractory participants (N = 25)	1 (4.00)	8 (32.00)	7 (28.0)	5 (20.0)	4 (16.0)

*Note.* Table format adapted from Leucht et al. [94]

PANSS: Positive and Negative Syndrome Scale. Refractory status was assessed using Kane’s criteria on treatment-resistance schizophrenia [123]

## Discussion

Given the significant individual and economic burden associated with exacerbations of psychotic disorders and hospitalisation, improving inpatient treatment is a critical concern for healthcare services [37]. An important contribution in this respect is the development of interventions targeting mechanisms of therapeutic change [97, 98] that are moreover adapted to the specific needs of acute inpatients [99]. The present study is the first exploratory study conducted within an acute psychiatric inpatient ward that investigates the feasibility, acceptability, and clinical outcomes of a mechanism-based and modularized group intervention targeting metacognitive change mechanisms in acute psychosis.

Results from the trial suggest that our group intervention was both feasible and acceptable, meeting the desired criteria for feasibility trials as outlined in guidelines [46, 100]. Despite COVID-19 pandemic-related challenges such as temporary closed wards and group format limitations, we recruited 37 participants within nine months, exceeding our pre-set recruitment target of 20 patients. Retention and attendance rates were both above the 80% benchmark, with overall satisfaction ratings exactly reaching the 80% acceptability target. The low dropout and missing data rates, and participants’ positive feedback in the questionnaires and interviews, further indicate high commitment and satisfaction with the treatment. Despite high symptom burden among participants with PANSS total scores comparable to average inpatients with acute psychosis [101, 102], there were no related adverse events, indicating the intervention's safety. Overall, our study results on feasibility and acceptability align with previous research, indicating that group psychological interventions are feasible, safe, and acceptable for inpatients with PSDs in acute care settings [16, 63, 103, 104]. This adds to the growing evidence contradicting the idea that psychotherapy is neither feasible nor helpful for this specific patient population [63].

Our LMMs moreover revealed promising results with medium-to-large effect sizes supporting hypothesised improvements on all secondary clinical measures. The decrease in negative symptoms is particularly noteworthy, as they greatly impair the functioning of those affected and have been reported to be resistant to pharmacotherapy and psychosocial treatments [105]. Participants in our study had significantly lower rehospitalisation rates compared to the average readmission rate of 50% within a year [106]. However, it's important to note that this interpretation is limited, as we only had access to readmission data from our hospital and not from other hospitals where patients may have been admitted during the follow-up period. The response rates in terms of PANSS reduction and CGI improvement moreover exceeded those of sole antipsychotic drug trials [102, 107], further supporting the potential clinical benefit of our mechanism-based intervention and meriting exploration in a larger scale study. Our findings are also consistent with above mentioned studies, which, next to demonstrating positive feasibility and acceptance, likewise presented preliminary encouraging results on clinical outcomes such as PANSS and WHODAS [16, 63, 103, 104].

Furthermore, our findings on assumed change mechanisms add support to the proof-of-concept of our underlying metacognitive treatment model. The post-Module-II improvements on cognitive insight measured with BCIS thereby are consistent with previous studies reporting immediate small post-intervention effects on self-certainty scores, with positive effects on self-reflectiveness showing only at the six-month follow-up [108, 109]. This suggests a previously discussed “sleeper” effect of MCT [110], that needs further exploration in future research studying long-term effects of cognitive insight [82, 108]. Significant post-Module-III reductions of cognitive fusion on the other hand are comparable to previous research reporting medium effect size changes in CFQ scores after four weeks of mindfulness-based group therapy for inpatients with PSDs [63]. Literature moreover discusses the mediating role of cognitive defusion in increasing psychological flexibility and thus fostering effective coping necessary for reducing symptom believability, subjective symptom severity, and psychosis-related distress in acute inpatients [111–113]. In summary, findings on potential change mechanisms underlying the respective modules were promising, but further exploration through mediation analyses in a randomized controlled trial is necessary before making viable statements [40, 114, 115].

### Strengths and limitations

The major strengths in our study included the adherence to a pre-registered trial protocol, pre-set feasibility benchmarks, the use of well-validated qualitative and quantitative assessments (rater and self-report), the detailed assessment of psychotropic medication, and use of complementary treatment elements to control for potential confounding variables. Moreover, the broad inclusion criteria (e.g. no restriction on substance abuse or ECT) allowed capturing a diverse range of patients that were actively involved in the intervention refinement through codesign activities during the whole study period [116]. In addition, the use of a contextualized, flexible (modularized) and targeted (change mechanisms) treatment approach allowed for individualized and tailored interventions, increasing the potential for positive treatment outcomes in acute inpatients with psychosis [36]. Finally, our LMM analyses captured the nested structure of our data and delivered more valid standard error estimates than common analysis of variance. In addition, we controlled for several confounders in our LMMs making our results on time effects on the outcome variables more reliable  and unbiased, despite the small sample size.

As an exploratory phase II study, there are several methodological limitations to consider. Firstly, the lack of a control group and the absence of restrictions on additional treatment modalities make it difficult to reliably estimate the intervention’s effectiveness. Despite controlling for covariates, preliminary clinical outcomes need to be viewed with caution since the intervention's effectiveness cannot be conclusively determined yet. Secondly, the assessments and therapy were mainly carried out by the same researchers. While assessments were strictly conducted according to protocol, this could have led to biases. Nevertheless, there was consistency in the effects observed between rater-based and self-report measures. Thirdly, the small sample size limits the statistical power of our LMMs, although it can be considered sufficient to answer the question of feasibility and acceptability. Fourthly, no follow-up measurements were included to test lasting treatment effects on secondary clinical outcomes and change mechanisms. Fifthly, the overall positive feedback given in the open-ended sections in the modules’ feedback questionnaires and the semi-structured interviews may be the result of a selection bias, as only patients who were already specifically “motivated” may have chosen to answer and/or to participate. Lastly, participants’ personal therapy goals (see Supplementary Table 5) did not always match group contents. However, personal topics were discussed in individual sessions and treatment personalization will be subject to further research.

Future research should adjust therapy contents and the study’s framework according to participants’ feedback and feasibility measures, including bigger sample sizes, blinded assessments, randomization, and an active control condition not focusing on the targeted change mechanisms to explore the treatment’s internal validity [115, 117] (see Supplementary Table 8 for planned adjustments). To provide further proof-of-concept for the metacognitive-based treatment model, additional mechanism measures should be added, such as direct measures of cognitive biases e.g. jumping to conclusion (JTC) bias [118] and theory of mind (ToM) impairments [119], along with mediation analyses and follow-up timepoints (also including information on readmissions to other hospitals) to examine the effects of change mechanisms [114, 115, 117, 120, 121]. The ultimate goal is to identify moderators of outcome to ensure the intervention is matched to the patient’s need and personal therapy goals, hence providing personalized treatment [42, 122] (for further details see Supplementary Table 8).

## Conclusion

Overall, the current results indicate that it is feasible and acceptable to conduct a mechanism-based and modularized group intervention focusing on metacognitive change mechanisms in acute psychiatric settings. The encouraging preliminary outcomes on clinical measures and change mechanisms moreover support the metacognitive treatment model. Further evaluation of the intervention and change mechanisms is warranted.

### Supplementary Information

Below is the link to the electronic supplementary material.

Supplementary file1 (DOCX 714 kb)

## Data Availability

Data contributing to the results are included in the article/Supplementary Material. Additional inquiries can be directed to the corresponding author.

## References

[1] Perälä J, Suvisaari J, Saarni SI (2007). Lifetime prevalence of psychotic and bipolar I disorders in a general population. Arch Gen Psychiatry.

[2] Chaiyakunapruk N, Chong HY, Teoh SL (2016). Global economic burden of schizophrenia: a systematic review. Neuropsychiatr Dis Treat.

[3] Cloutier M, Aigbogun MS, Guerin A (2016). The economic burden of schizophrenia in the United States in 2013. J Clin Psychiatry.

[4] Frey S (2014). The economic burden of schizophrenia in Germany: a population-based retrospective cohort study using genetic matching. Eur Psychiatr.

[5] van der Post LF, Peen J, Visch I (2014). Patient perspectives and the risk of compulsory admission: the Amsterdam Study of Acute Psychiatry V. Int J Soc Psychiatry.

[6] Priebe S, Badesconyi A, Fioritti A (2005). Reinstitutionalisation in mental health care: comparison of data on service provision from six European countries. BMJ.

[7] Bowers L, Jeffery D, Bilgin H (2008). Psychiatric Intensive Care Units: a Literature Review. Int J Soc Psychiatry.

[8] Wood L, Alsawy S (2016). Patient experiences of psychiatric inpatient care: a systematic review of qualitative evidence. J Psych Intensive Care.

[9] NICE (2014) Psychosis and schizophrenia in adults: prevention and management. National Institute of Clinical Excellence, London 32207892

[10] Gaebel W, Hasan A, Falkai P (2019). S3-Leitlinie Schizophrenie.

[11] Gaudiano BA, Herbert JD (2006). Acute treatment of inpatients with psychotic symptoms using Acceptance and Commitment Therapy: pilot results. Behav Res Ther.

[12] Wood L, Williams C, Billings J, Johnson S (2019). The role of psychology in a multidisciplinary psychiatric inpatient setting: perspective from the multidisciplinary team. Psychol Psychother Theory Res Pract.

[13] Barnicot K, Michael C, Trione E (2020). Psychological interventions for acute psychiatric inpatients with schizophrenia-spectrum disorders: a systematic review and meta-analysis. Clin Psychol Rev.

[14] Wood L, Williams C, Billings J, Johnson S (2020). A systematic review and meta-analysis of cognitive behavioural informed psychological interventions for psychiatric inpatients with psychosis. Schizophr Res.

[15] Jacobsen P, Hodkinson K, Peters E, Chadwick P (2018). A systematic scoping review of psychological therapies for psychosis within acute psychiatric in-patient settings. Br J Psychiatry.

[16] Aghotor J, Pfueller U, Moritz S (2010). Metacognitive training for patients with schizophrenia (MCT): feasibility and preliminary evidence for its efficacy. J Behav Ther Exp Psychiatry.

[17] Schaeuffele C, Schulz A, Knaevelsrud C (2021). CBT at the crossroads: the rise of transdiagnostic treatments. J Cogn Ther.

[18] Hayes SC, Hofmann SG (2021). “Third-wave” cognitive and behavioral therapies and the emergence of a process-based approach to intervention in psychiatry. World Psychiatry.

[19] Lysaker PH, Gagen E, Moritz S, Schweitzer R (2018). Metacognitive approaches to the treatment of psychosis: a comparison of four approaches. PRBM.

[20] Moritz S, Lysaker PH (2018). Metacognition—what did James H. Flavell really say and the implications for the conceptualization and design of metacognitive interventions. Schizophr Res.

[21] Flavell JH (1979). Metacognition and cognitive monitoring: a new area of cognitive-developmental inquiry. Am Psychol.

[22] Moritz S, Woodward TS (2007). Metacognitive training in schizophrenia: from basic research to knowledge translation and intervention. Curr Opin Psychiatry.

[23] Kumar D, Menon M, Moritz S, Woodward TS (2015). Using the back door: Metacognitive training for psychosis. Psychosis.

[24] Moritz S, Veckenstedt R, Bohn F (2013). Complementary group Metacognitive Training (MCT) reduces delusional ideation in schizophrenia. Schizophr Res.

[25] Morris EMJ, Johns LC, Oliver JE (2013). Acceptance and commitment therapy and mindfulness for psychosis.

[26] Bernstein A, Hadash Y, Lichtash Y (2015). Decentering and related constructs: a critical review and metacognitive processes model. Perspect Psychol Sci.

[27] Assaz DA, Roche B, Kanter JW, Oshiro CKB (2018). Cognitive defusion in acceptance and commitment therapy: what are the basic processes of change?. Psychol Rec.

[28] Bacon T, Farhall J, Fossey E (2014). The active therapeutic processes of acceptance and commitment therapy for persistent symptoms of psychosis: clients’ perspectives. Behav Cogn Psychother.

[29] Bach P, Gaudiano BA, Hayes SC, Herbert JD (2013). Acceptance and commitment therapy for psychosis: intent to treat, hospitalization outcome and mediation by believability. Psychosis.

[30] Tyrberg MJ, Carlbring P, Lundgren T (2017). Brief acceptance and commitment therapy for psychotic inpatients: a randomized controlled feasibility trial in Sweden. Nordic Psychol.

[31] Lysaker PH, Hamm JA, Hasson-Ohayon I (2018). Promoting recovery from severe mental illness: implications from research on metacognition and metacognitive reflection and insight therapy. WJP.

[32] Lysaker PH, Gagen E, Klion R (2020). Metacognitive reflection and insight therapy: a recovery-oriented treatment approach for psychosis. PRBM.

[33] Inchausti F, García-Mieres H, García-Poveda NV (2023). Recovery-focused metacognitive interpersonal therapy (MIT) for adolescents with first-episode psychosis. J Contemp Psychother.

[34] Hayes SC, Strosahl K, Wilson KG (2016) Acceptance and commitment therapy: the process and practice of mindful change. Guilford, New York

[35] Wells A (2011). Metacognitive therapy for anxiety and depression.

[36] Gussmann E, Lucae S, Falkai P (2023). Developing a mechanism-based therapy for acute psychiatric inpatients with psychotic symptoms: an Intervention Mapping approach. Front Psychiatry.

[37] Wood L, Williams C, Billings J, Johnson S (2019). The therapeutic needs of psychiatric in-patients with psychosis: a qualitative exploration of patient and staff perspectives. BJPsych Open.

[38] Bartholomew Eldredge LK (2016). Planning health promotion programs: an intervention mapping approach.

[39] Bleijenberg N, de Man-van Ginkel JM, Trappenburg JCA (2018). Increasing value and reducing waste by optimizing the development of complex interventions: enriching the development phase of the Medical Research Council (MRC) Framework. Int J Nurs Stud.

[40] Kazdin AE (2007). Mediators and mechanisms of change in psychotherapy research. Annu Rev Clin Psychol.

[41] Hofmann SG, Hayes SC (2019). The future of intervention science: process-based therapy. Clin Psychol Sci.

[42] Elsaesser M, Herpertz S, Piosczyk H (2022). Modular-based psychotherapy (MoBa) versus cognitive–behavioural therapy (CBT) for patients with depression, comorbidities and a history of childhood maltreatment: study protocol for a randomised controlled feasibility trial. BMJ Open.

[43] Philippot P, Bouvard M, Baeyens C, Dethier V (2019). Case conceptualization from a process-based and modular perspective: rationale and application to mood and anxiety disorders. Clin Psychol Psychother.

[44] Walser RD, O’Connell M (2021). Acceptance and commitment therapy and the therapeutic relationship: rupture and repair. J Clin Psychol.

[45] Newton E, Larkin M, Melhuish R, Wykes T (2007). More than just a place to talk: young people’s experiences of group psychological therapy as an early intervention for auditory hallucinations. Psychol Psychother Theory Res Pract.

[46] Avery KNL, Williamson PR, Gamble C (2017). Informing efficient randomised controlled trials: exploration of challenges in developing progression criteria for internal pilot studies. BMJ Open.

[47] Beck AK, Baker A, Jones S (2018). Exploring the feasibility and acceptability of a recovery-focused group therapy intervention for adults with bipolar disorder: trial protocol. BMJ Open.

[48] Waller H, Emsley R, Freeman D (2015). Thinking well: a randomised controlled feasibility study of a new CBT therapy targeting reasoning biases in people with distressing persecutory delusional beliefs. J Behav Ther Exp Psychiatry.

[49] Morrison AP, Pyle M, Chapman N (2014). Metacognitive therapy in people with a schizophrenia spectrum diagnosis and medication resistant symptoms: a feasibility study. J Behav Ther Exp Psychiatry.

[50] Forkert A, Brown P, Freeman D, Waite F (2022). A compassionate imagery intervention for patients with persecutory delusions. Behav Cogn Psychother.

[51] Lancaster GA, Thabane L (2019). Guidelines for reporting non-randomised pilot and feasibility studies. Pilot Feasibility Stud.

[52] Eldridge SM, Lancaster GA, Campbell MJ (2016). Defining feasibility and pilot studies in preparation for randomised controlled trials: development of a conceptual framework. PLoS ONE.

[53] Jelinek L, Hauschildt M, Moritz S (2015) Metakognitives Training bei Depression (D-MKT): mit E-Book inside und Trainingsmaterial. Beltz, Weinheim

[54] Fischer R, Scheunemann J, Bohlender A (2022). ‘You are trying to teach us to think more slowly!’: adapting metacognitive training for the acute care setting—a case report. Clin Psychol Psychoth.

[55] Carballedo A, Doyle M (2011). Criteria for compulsory admission in some European countries. Int psychiatry.

[56] Roth A, Pilling S (2012) A competence framework for psychological interventions with people with psychosis and bipolar disorder. University College London, London

[57] Baigent M (2012). Managing patients with dual diagnosis in psychiatric practice. Curr Opin Psychiatry.

[58] Moritz S, Vitzthum F, Randjbar S (2010). Detecting and defusing cognitive traps: metacognitive intervention in schizophrenia. Curr Opin Psychiatry.

[59] Linden M (2013). How to define, find and classify side effects in psychotherapy: from unwanted events to adverse treatment reactions: side effects in psychotherapy: the UE-ATR checklist. Clin Psychol Psychother.

[60] Eldridge SM, Chan CL, Campbell MJ (2016). CONSORT 2010 statement: extension to randomised pilot and feasibility trials. BMJ.

[61] Moritz S, Woodward T (2007). Metacognitive training for schizophrenia patients (MCT): a pilot study on feasibility, treatment adherence, and subjective efficacy. German J Psychiatry.

[62] Thabane L, Ma J, Chu R (2010). A tutorial on pilot studies: the what, why and how. BMC Med Res Methodol.

[63] Böge K, Hahne I, Bergmann N (2021). Mindfulness-based group therapy for in-patients with schizophrenia spectrum disorders—feasibility, acceptability, and preliminary outcomes of a rater-blinded randomized controlled trial. Schizophr Res.

[64] Wood L, Williams C, Pinfold V (2022). Crisis-focused Cognitive Behavioural Therapy for psychosis (CBTp) in acute mental health inpatient settings (the CRISIS study): protocol for a pilot randomised controlled trial. Pilot Feasibility Stud.

[65] Lewis M, Bromley K, Sutton CJ (2021). Determining sample size for progression criteria for pragmatic pilot RCTs: the hypothesis test strikes back!. Pilot Feasibility Stud.

[66] Kay SR, Fiszbein A, Opler LA (1987). The positive and negative syndrome scale (PANSS) for schizophrenia. Schizophr Bull.

[67] Kay SR, Opler LA, Lindenmayer J-P (1988). Reliability and validity of the positive and negative syndrome scale for schizophrenics. Psychiatry Res.

[68] Haddock G, McCarron J, Tarrier N, Faragher EB (1999). Scales to measure dimensions of hallucinations and delusions: the psychotic symptom rating scales (PSYRATS). Psychol Med.

[69] Hall RCW (1995). Global assessment of functioning. Psychosomatics.

[70] Pedersen G, Urnes Ø, Hummelen B (2018). Revised manual for the Global Assessment of Functioning scale. Eur psychiatr.

[71] Grootenboer EMV, Giltay EJ, van der Lem R (2012). Reliability and validity of the Global Assessment of Functioning Scale in clinical outpatients with depressive disorders: GAF in outpatients with depression. J Eval Clin Pract.

[72] Guy W (1976) Clinical Global Impressions. ECDEU assessment manual for psychopharmacology. National Institute of Mental Health, Rockville

[73] Busner J, Targum SD (2007). The clinical global impressions scale: applying a research tool in clinical practice. Psychiatry (Edgmont).

[74] Leucht S, Kane JM, Etschel E (2006). Linking the PANSS, BPRS, and CGI: clinical implications. Neuropsychopharmacology.

[75] Ustun TB, Chatterji S, Kostanjsek N (2010). Developing the world health organization disability assessment schedule 2.0. Bull World Health Organ.

[76] World Health Organization (2001) International classification of functioning, disability and health : ICF. World Health Organization, Geneva

[77] Holmberg C, Gremyr A, Torgerson J, Mehlig K (2021). Clinical validity of the 12-item WHODAS-2.0 in a naturalistic sample of outpatients with psychotic disorders. BMC Psychiatry.

[78] Mas-Expósito L, Amador-Campos JA, Gómez-Benito J, Lalucat-Jo L (2012). The World Health Organization Short Disability Assessment Schedule: a validation study in patients with schizophrenia. Compr Psychiatry.

[79] Sabbag S, Twamley EW, Vella L (2012). Predictors of the accuracy of self assessment of everyday functioning in people with schizophrenia. Schizophr Res.

[80] Gspandl S, Peirson RP, Nahhas RW (2018). Comparing global assessment of functioning (GAF) and World Health Organization Disability Assessment Schedule (WHODAS) 2.0 in schizophrenia. Psychiatry Res.

[81] Ustun TB, Kostanjesek N, Chatterji S, Rehm J, World Health Organization (eds) (2010) Measuring health and disability: manual for WHO Disability Assessment Schedule (WHODAS 2.0), p 88

[82] Beck A (2004). A new instrument for measuring insight: the Beck Cognitive Insight Scale. Schizophr Res.

[83] China C, Hansen LB, Gillanders DT, Benninghoven D (2018). Concept and validation of the German version of the Cognitive Fusion Questionnaire (CFQ-D). J Contextual Behav Sci.

[84] Gillanders DT, Bolderston H, Bond FW (2014). The development and initial validation of the cognitive fusion questionnaire. Behav Ther.

[85] Braun V, Clarke V (2006). Using thematic analysis in psychology. Qual Res Psychol.

[86] Leucht S, Samara M, Heres S, Davis JM (2016). Dose equivalents for antipsychotic drugs: the DDD method: table 1. SCHBUL.

[87] Sommet N, Morselli D (2021). Keep calm and learn multilevel linear modeling: a three-step procedure using SPSS, Stata, R, and Mplus. Int Rev Soc Psychol.

[88] Kreft I, de Leeuw J (1998). Introducing multilevel modeling.

[89] Snijders TAB, Bosker RJ (2012). Multilevel analysis: an introduction to basic and advanced multilevel modeling.

[90] Nakagawa S, Schielzeth H (2013). A general and simple method for obtaining R2 from generalized linear mixed-effects models. Methods Ecol Evol.

[91] Bell A, Fairbrother M, Jones K (2019). Fixed and random effects models: making an informed choice. Qual Quant.

[92] Oberauer K (2022). The importance of random slopes in mixed models for bayesian hypothesis testing. Psychol Sci.

[93] Moritz S, Menon M, Andersen D (2018). Moderators of symptomatic outcome in metacognitive training for psychosis (MCT). Who benefits and who does not?. Cogn Ther Res.

[94] Leucht S, Davis JM, Engel RR (2009). Definitions of response and remission in schizophrenia: recommendations for their use and their presentation. Acta Psychiatr Scand.

[95] Leucht S, Davis JM, Engel RR (2007). Defining ‘response’ in antipsychotic drug trials: recommendations for the use of scale-derived cutoffs. Neuropsychopharmacol.

[96] R Core Team (2021) R: a language and environment for statistical computing. R Foundation for Statistical Computing, Vienna

[97] Garety PA, Freeman D (2013). The past and future of delusions research: from the inexplicable to the treatable. Br J Psychiatry.

[98] Freeman D (2011). Improving cognitive treatments for delusions. Schizophr Res.

[99] Wood L, Williams C, Billings J, Johnson S (2019). Psychologists’ perspectives on the implementation of psychological therapy for psychosis in the acute psychiatric inpatient setting. Qual Health Res.

[100] Thabane L, Lancaster G (2019). A guide to the reporting of protocols of pilot and feasibility trials. Pilot Feasibility Stud.

[101] Möller H-J, Bäuml J, Ferrero F (1997). Risperidone in the treatment of schizophrenia: results of a study of patients from Germany, Austria, and Switzerland. Eur Arch Psychiatry Clin Neurosci.

[102] Chouinard G, Jones B, Remington G (1993). A Canadian multicenter placebo-controlled study of fixed doses of risperidone and haloperidol in the treatment of chronic schizophrenic patients. J Clin Psychopharmacol.

[103] Gaudiano BA, Ellenberg S, Ostrove B (2020). Feasibility and preliminary effects of implementing acceptance and commitment therapy for inpatients with psychotic-spectrum disorders in a clinical psychiatric intensive care setting. J Cogn Psychother.

[104] Jacobsen P, Peters E, Robinson EJ, Chadwick P (2020). Mindfulness-based crisis interventions (MBCI) for psychosis within acute inpatient psychiatric settings; a feasibility randomised controlled trial. BMC Psychiatry.

[105] McCutcheon RA, Reis Marques T, Howes OD (2020). Schizophrenia—an overview. JAMA Psychiat.

[106] Kissling W (1991). The current unsatisfactory state of relapse prevention in schizophrenic psychoses–suggestions for improvement. Clin Neuropharmacol.

[107] Marder SR, Meibach RC (1994). Risperidone in the treatment of schizophrenia. Am J Psychiatry.

[108] Ochoa S, López-Carrilero R, Barrigón ML (2017). Randomized control trial to assess the efficacy of metacognitive training compared with a psycho-educational group in people with a recent-onset psychosis. Psychol Med.

[109] Birulés I, López-Carrilero R, Cuadras D (2020). Cognitive insight in first-episode psychosis: changes during metacognitive training. JPM.

[110] Moritz S, Veckenstedt R, Andreou C (2014). Sustained and “Sleeper” effects of group metacognitive training for schizophrenia: a randomized clinical trial. JAMA Psychiat.

[111] Gaudiano BA, Herbert JD, Hayes SC (2010). Is it the symptom or the relation to it? Investigating potential mediators of change in acceptance and commitment therapy for psychosis. Behav Ther.

[112] Jansen JE, Gleeson J, Bendall S (2020). Acceptance- and mindfulness-based interventions for persons with psychosis: a systematic review and meta-analysis. Schizophr Res.

[113] Lee JW, Park HS (2018). Development and effects of an acceptance commitment-based cognitive behavioral program for patients with schizophrenia. J Korean Acad Psychiatr Ment Health Nurs.

[114] Schlier B, Ludwig L, Wiesjahn M (2020). Fostering coping as a mechanism of symptom change in cognitive behavioural therapy for psychosis. Schizophr Res.

[115] Garety P, Waller H, Emsley R (2015). Cognitive mechanisms of change in delusions: an experimental investigation targeting reasoning to effect change in paranoia. Schizophr Bull.

[116] O’Cathain A, Croot L, Sworn K (2019). Taxonomy of approaches to developing interventions to improve health: a systematic methods overview. Pilot Feasibility Stud.

[117] Freeman D, Dunn G, Startup H (2015). Effects of cognitive behaviour therapy for worry on persecutory delusions in patients with psychosis (WIT): a parallel, single-blind, randomised controlled trial with a mediation analysis. The Lancet Psychiatry.

[118] Moritz S, Woodward TS (2006). A generalized bias against disconfirmatory evidence in schizophrenia. Psychiatry Res.

[119] Yeh Y-C, Lin C-Y, Li P-C (2021). A systematic review of the current measures of theory of mind in adults with schizophrenia. IJERPH.

[120] Pine JG, Moe AM, Wastler HM, Breitborde NJK (2022). Improvements in metacognition mediate the effect of metacognitive remediation therapy: A non-randomized controlled study among individuals with first-episode psychosis. Early Intervention Psych.

[121] Thomas N, Farhall J, Foley F (2016). Randomised controlled trial of a digitally assisted low intensity intervention to promote personal recovery in persisting psychosis: SMART-Therapy study protocol. BMC Psychiatry.

[122] Hazell CM, Hayward M, Cavanagh K, Strauss C (2016). A systematic review and meta-analysis of low intensity CBT for psychosis. Clin Psychol Rev.

[123] Kane JM, Agid O, Baldwin ML (2019). Clinical guidance on the identification and management of treatment-resistant schizophrenia. J Clin Psychiatry.

