# Supplementary material for: Targeting metacognitive change mechanisms in acute inpatients with psychotic symptoms: feasibility and acceptability of a modularized group intervention
Source: Eur Arch Psychiatry Clin Neurosci. 2023 Sep 23;274(4):963–79. doi: 10.1007/s00406-023-01690-y (PMC11127867; doi:10.1007/s00406-023-01690-y)
Supplement: Supplementary file 1 — Supplementary file1 (DOCX 714 kb) [file 406_2023_1690_MOESM1_ESM.docx]

Online Supplementary Material

Targeting metacognitive change mechanisms in acute inpatients with psychotic symptoms: Feasibility and acceptability of a modularized group intervention

Eva Gussmann^1*^, Christoph Lindner^1^, Susanne Lucae^1,3^, Peter Falkai^1,3^, Frank Padberg^3^, Samy Egli^1^, Johannes Kopf-Beck^1,2^

^1^ Max Planck Institute of Psychiatry, Munich, Germany

^2^ Faculty of Psychology and Educational Sciences, Department of Psychology, Ludwig Maximilian University, Munich, Germany

^3^ Department of Psychiatry and Psychotherapy, University Hospital, LMU Munich, Germany

*** Correspondence:**Eva Gussmann, M.Sc.
eva_gussmann@psych.mpg.de

**Table of Contents**

Supplementary Methods 3

Supplementary Methods 1. Background to the mechanism-based group therapy concept for the acute psychiatric inpatient ward 3

Supplementary Methods 2. Routine care on the acute psychiatric inpatient ward of the Max-Planck-Institute of Psychiatry in Munich, Germany 4

Supplementary Methods 3. Feedback questionnaire for the Module Defusion 5

Supplementary Methods 4. Semi-structured interview conducted at the end of the intervention with selected participants 6

Supplementary Figures 7

Supplementary Figure 1. Mechanism-based group therapy concept on the acute psychiatric inpatient ward 7

Supplementary Figure 2. Problem model of severe psychotic symptoms, danger to self and others and hospitalisation 8

Supplementary Figure 3. Underlying therapeutic model of the mechanism-based group intervention 9

Supplementary Figure 3. Thematic analysis of themes and sub-themes for “positive group aspects” in the semi-structured interviews 10

Supplementary Figure 4. Thematic analysis of themes and sub themes for “insights from group therapy” in the semi-structured interviews 11

Supplementary Figure 5. Thematic analysis of themes and sub themes for “negative group aspects” in the semi-structured interviews 12

Supplementary Figure 6. Thematic analysis of themes and sub themes for “study setup” in the semi-structured interviews 13

Supplementary Tables 14

Supplementary Table 1. Overview of intervention’s objectives and core exercises 14

Supplementary Table 2. Participants’ attendance rates and reasons for non-attendance 16

Supplementary Table 3. Participants’ qualitative feedback on the feedback questionnaires of each module and the overall intervention 17

Supplementary Table 4. Participation in supplementary treatments additionally to the experimental group therapy 19

Supplementary Table 5. Content of individual psychotherapy sessions during the study period 20

Supplementary Table 6. Transcript notes of individual quotes supporting the thematic analysis for each question in the semi-structured interview 24

Supplementary Table 7. Codes identified for each participant for the thematic analysis of the semi-structured interview 26

Supplementary Table 8. Recommendations for future research 30

References 33

# Supplementary Methods

## Supplementary Methods 1. Background to the mechanism-based group therapy concept for the acute psychiatric inpatient ward

The experimental mechanism-based group therapy for inpatients with acute psychosis was part of an already established modular and mechanism-based psychosocial group therapy concept on the acute psychiatric inpatient ward. The existing concept thereby covered the change mechanisms of distress tolerance, impulsivity reduction, behavioural activation, information processing, and self-management, which have been identified as relevant treatment targets in acute psychiatric settings [1, 2]. Based on this selection and with reference to evidence-based treatment manuals, we implemented four group modules, including a transdiagnostic Skillstraining, a transdiagnostic Resource Group, a transdiagnostic Psychoeducation Group and a transdiagnostic Crisis-Competence-Group (see Supplementary Figure 1). All group modules were adapted to a crisis-focused setting by being brief, easy to understand and coping-oriented [3].

Due to the lack of available treatments specifically tailored for inpatients with acute psychotic symptoms [4], a new mechanism-based group was developed (see Supplementary Figure 1). As there was limited evidence available for existing concepts in this patient population and setting, a rigorous scientific process was followed during the intervention design [5] and feasibility study [6], which we described in our previous [7] and current work. Our target inpatient group for the design and evaluation study encompassed the entire psychosis-spectrum, including affective disorders with psychotic symptoms and comorbid diagnosis. However, we ensured that the group was also designed using transdiagnostic principles to allow for future expansion to other patient populations.

Our feasibility study resembles an early Phase II clinical trial with the aim of testing the feasibility, acceptability and safety of the novel intervention with a small amount of diverse participants [8, 9]. Given that the group treatment is feasible and safe, we plan to further study the effect of hypothesized change mechanisms with the help of mediation analyses in Phase III and IV studies. Moreover, our goal is to further evaluate which patients will particularly benefit from the concept by studying moderators [10]. Therewith, our ultimate goal is to personalize treatment and optimize outcomes by a) identifying patients’ relevant change mechanisms right on admission, b) creating a treatment plan that combines interventions targeting various key change mechanisms, and c) offering treatment in different therapeutic modes e.g. individual and group therapy to meet patients’ needs (see Supplementary Figure 1) [11–13].

## Supplementary Methods 2. Routine care on the acute psychiatric inpatient ward of the Max-Planck-Institute of Psychiatry in Munich, Germany

1. **Target patient group and treatment mission**

24 available beds for individuals with severe symptoms and acute crises. The treatment spectrum covers all diagnoses with a particular focus on psychosis-spectrum disorders. Treatment goals include detailed differential diagnostic assessment, medication adjustment, crisis intervention, reinforcement of coping strategies, family involvement, psychosocial counselling and referral, and discharge management. Psychiatric detention may be ordered to protect patients from danger to themselves or others.

1. **Staffing on the acute psychiatric inpatient ward**

- Two to three nurses each shift (morning, day, night)
- One senior physician and three residents
- One psychologist trained in CBT
- One social worker, one occupational therapist
- Work for different wards: Nutritionist, physiotherapist, internist, and sports therapist

1. **Treatment options on the acute psychiatric inpatient ward**

- Neurological assessment (i.e. MRI, Computed tomography, lumbar puncture)
- (Psychotropic) medication
- Electroconvulsive therapy
- Transcranial magnetic stimulation
- Physician’s consultation (25 minutes per week)
- Senior physician rounds (15 minutes per week)
- Individual psychotherapy (25 to 50 minutes per week)
- Group psychotherapy (50-100 minutes per week)
- Social counseling
- Occupational therapy
- Sports therapy
- Optional: Nutritional counseling and physical therapy

## Supplementary Methods 3. Feedback questionnaire for the Module Defusion

**Feedback questionnaire Module *Defusion***

Study-ID: Date:

How did you experience the last four therapy sessions on the Defusion module? Please indicate how much the following 16 statements apply to you according to the rating scale shown below. Please edit all statements, even if some of the content may not seem entirely appropriate to you.

**0 = Does not apply at all**; 1 = Applies to a small extent; 2 = Applies to some extent; 3 = Applies to a great extend; **4 = Applies exactly**

| 1. I found the Defusion module useful and helpful. | 0 1 2 3 4 |
| --- | --- |
| 1. I was able to understand the contents of the module well. | 0 1 2 3 4 |
| 1. I can apply the contents of the module well in my everyday life. | 0 1 2 3 4 |
| 1. The module has given me suggestions on how to cope with my complaints. | 0 1 2 3 4 |
| 1. The objectives of the module are clear to me. | 0 1 2 3 4 |
| 1. I had fun in the sessions. | 0 1 2 3 4 |
| 1. I would rather spend my time elsewhere than in group therapy. | 0 1 2 3 4 |
| 1. I think it is good that the therapy takes place in the group. | 0 1 2 3 4 |
| 1. I felt comfortable in the group. | 0 1 2 3 4 |
| 1. After this module I think that this form of therapy is promising for my treatment. | 0 1 2 3 4 |
| 1. I would recommend this module to other patients. | 0 1 2 3 4 |
| 1. Overall I am satisfied with the Defusion module. | 0 1 2 3 4 |

1. What do you think about the number of sessions in the Defusion module (4 sessions)?
2. Too few b) just right c) too many
3. What do you think about the duration of the sessions (60 min.)?
4. Too short b) just right c) too long
5. What did you personally take away from the Defusion module?

__________________________________________________________

1. Which topics that you find important have not been taken into account enough?

__________________________________________________________

## Supplementary Methods 4. Semi-structured interview conducted at the end of the intervention

## with selected participants

**Semi-structured interview to explore patients’ subjective experience of**

**the group therapy**

**Main questions**

*(Prompts are in italics)*

1. Can you tell me about what you liked about our group therapy?

- *What was it like taking part?*
- *Did you feel comfortable in the group?*
- *Did you like the setting and kind of exercises?*
- *Did the main topic of the group appeal to you?*

1. What insights and strategies for dealing with your thoughts will you take away from the group?

- *What have you learned in general about your thoughts?*
- *Are there any particular exercises you liked?*
- *Are you already using new strategies in dealing with your thoughts?*

1. Was there anything you didn’t like about the group?

- *Did you have difficulties following the topics?*
- *Did you have difficulties joining the exercises?*
- *Would you rather have participated in a different group?*

1. How do you evaluate the framework of the group therapy?

- *How do you rate the frequency and duration of the group sessions?*
- *How do you rate the frequency and effort of the questionnaires?*
- *Any suggestions for the future? Anything else you would like to add?*

# Supplementary Figures

## Supplementary Figure 1. Mechanism-based group therapy concept on the acute psychiatric inpatient ward

*Note.* Reprinted from “Developing a mechanism-based therapy for acute psychiatric inpatients with psychotic symptoms: An Intervention Mapping approach” [7]. Psychosocial treatment components present the targeted change mechanism with the name of the respective group/treatment module in square brackets. Existing mechanism-based groups on the acute psychiatric inpatient ward include a) a transdiagnostic Skillstraining (in total three sessions covering psychoeducation on tension regulation, testing of different stress-tolerance-skills, development of emergency plans and skill chains), b) a transdiagnostic Resource Group (in total three sessions covering psychoeducation on depression upward- and downward-spiral, development of positive activities and resources, day and week planning), c) a transdiagnostic Crisis-Competence Group (in total four sessions covering crisis formulation, early warning signs and coping strategies, emergency plan and discharge planning, and d) a transdiagnostic Psychoeducation Group (in total three sessions covering information on diathesis-stress-model, medication, and treatment options). All groups were adapted from existing group manuals [14–17] to fit the acute inpatient setting. Each group session lasts 50 minutes and takes place weekly. Inpatients are able to participate in two group therapies with the option for individual therapy. The experimental mechanism-based group therapy (in blue) was specifically designed for inpatients with acute psychotic symptoms and takes place twice a week with a total of nine sessions. The ultimate goal of the mechanism-based concept is to individually tailor treatment for acute inpatients by allocating them to the group therapies most likely to target individually relevant change mechanisms and personal preferences.

## Supplementary Figure 2. Problem model of severe psychotic symptoms, danger to self and others and hospitalisation


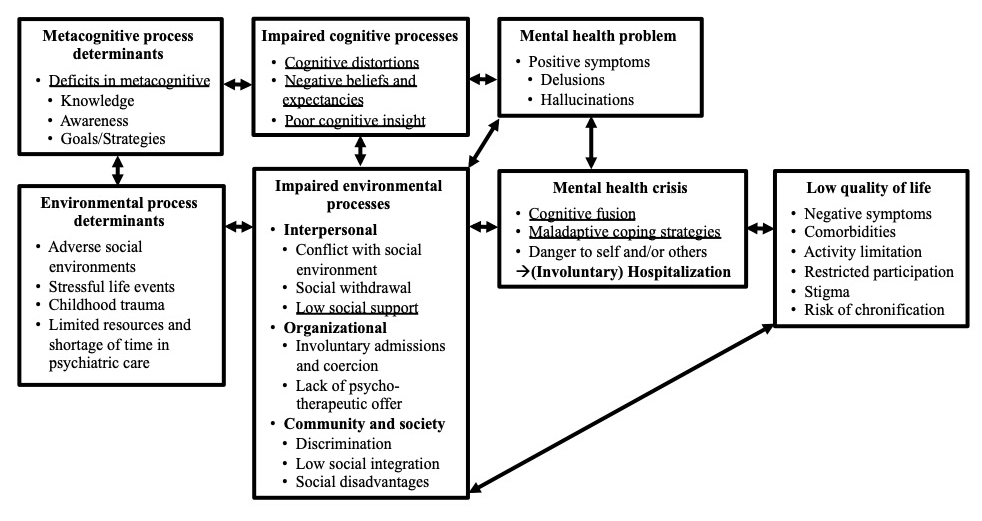


*Note.* Reprinted from “Developing a mechanism-based therapy for acute psychiatric inpatients with psychotic symptoms: An Intervention Mapping approach” [7]. Logical model of the problem of severe psychotic symptoms, danger to self and others, (involuntary) hospitalization and a resulting low quality of life. The model has a focus on psychological and social factors in the development of acute psychosis and does not consider biological factors e.g. genetics [18–22]. It moreover does not map the moderating or mediating relationships between variables, but rather aims to visualize the variability of factors and impaired processes that contribute to the main problems. Impaired processes that were identified as target areas for the underlying therapeutic model are underlined.

## Supplementary Figure 3. Underlying therapeutic model of the mechanism-based group intervention

*Note.* Reprinted from “Developing a mechanism-based therapy for acute psychiatric inpatients with psychotic symptoms: An Intervention Mapping approach” [7]. Underlying therapeutic change model showing what change is needed to manage severe psychotic symptoms acute crises. It points out the metacognitive change domains and belonging change mechanisms expected to influence the cognitive, behavioural and environmental outcomes that are in turn believed to improve mental health and quality of life. Hypothesized underlying target change mechanisms are put into square brackets. Regarding different psychotic symptoms, the intervention employs a cognitive perspective with auditory hallucinations being viewed as intrusive thoughts amplified and externalized through cognitive dissonance [23]. Hence, both delusional thoughts and hallucinations are referred to as distressing internal experiences, which generally creates a destigmatizing and normalizing therapy language [24].

## Supplementary Figure 3. Thematic analysis of themes and sub-themes for “positive group aspects” in the semi-structured interviews

*Note.* Thematic analysis for participants’ answers on the question “Can you tell me about what you liked about our group therapy?” in the semi-structured interview (see Supplementary Methods 4) revealed two themes with subthemes (see Supplementary Tables 6 and 7). Topics included helpful therapy contents (Defusion techniques, Coping strategies and Mindfulness) and supporting environment (Clear structure, Positive group atmosphere, and Information supply).

## Supplementary Figure 4. Thematic analysis of themes and sub themes for “insights from group therapy” in the semi-structured interviews

*Note.* Thematic analysis for participants’ answers on the question “What insights and strategies for dealing with your thoughts will you take away from the group?” in the semi-structured interview (see Supplementary Methods 4) revealed themes and subthemes on metacognitive knowledge and awareness (Awareness of thought patters and Identifying relation responses) and metacognitive strategies (Directing attention, Mindfulness, Disrupting thought-action and Recontextualization) (see Supplementary Tables 6 and 7).

## Supplementary Figure 5. Thematic analysis of themes and sub themes for “negative group aspects” in the semi-structured interviews

*Note.* Thematic analysis for participants’ answers on the question “Was there anything you didn’t like about the group?” in the semi-structured interview (see Supplementary Methods 4) included tight session schedules, partial content overload, too few practical exercises and a lack of motivation from fellow participants (see Supplementary Tables 6 and 7).

## Supplementary Figure 6. Thematic analysis of themes and sub themes for “study setup” in the semi-structured interviews

*Note.* Thematic analysis for participants’ answers on the question “How do you evaluate the framework of the group therapy?” in the semi-structured interview (see Supplementary Methods 4) comprised reducing group size, shortening session duration, and simplifying feedback questionnaires (see Supplementary Tables 6 and 7).

# Supplementary Tables

## Supplementary Table 1. Overview of intervention’s objectives and core exercises

| **Session** | **Title, main objective and target change mechanism** | **Core exercises and metaphors** |
| --- | --- | --- |
| 1 | **Psychoeducation**  Objective: Understanding the cognitive model, awareness of problematic cognitive biases and over identification/reaction to them  Target mechanism: Knowledge increase | Developing theory based on an everyday example (“Imagine your friend doesn’t call on your birthday”) and interactive group discussion  Source: MCT for depression [25] |
| **Module Cognitive Insight** [Metacognitive knowledge and awareness] | | |
| 2 | **Finding explanations**  Objective: Changing dysfunctional attributional patterns by understanding that multiple factors can lead to a scenario  Target mechanism: Attributional reasoning | Contemplating different causes for everyday examples and discussing negative consequences of monocausal attributions  Source: MCT for psychosis and MCT-acute [26, 27] |
| 3 | **Jumping to conclusions**  Objective: Avoiding premature first impressions, adjusting conclusion when new information emerges  Target mechanism: Interpretative reasoning | Holding back and revising premature decisions with the help of various fragmented picture tasks where patients have to guess the object behind it  Source: MCT for psychosis and MCT-acute [26, 27] |
| 4 | **To empathize**  Objective: Understanding that facial expressions can easily be misinterpreted, considering various information sources when assessing your opposite  Target mechanism: Social reasoning | Trying to guess what a person may feel or intends to do by judging pictures of their faces and discussing everyday examples  Source: MCT for psychosis and MCT-acute [26, 27] |
| 5 | **Mood and self-esteem**  Objective: Recognizing dysfunctional thinking styles, finding alternative views and engaging in positive actions  Target mechanism: Cognitive reappraisal | Gathering symptoms of depression, finding more helpful thoughts for negative cognitive schemas in various everyday examples, collecting positive activities to counteract depressive mood and low self-esteem  Source: MCT for psychosis and MCT-acute [26, 27] |
| **Module Cognitive Defusion** [Metacognitive goals and strategies] | | |
| 6 | **Noticing thoughts**  Objective: Being more present in the moment, noticing inner and outer sensations and responding more consciously to them  Target mechanism: Mindfulness | Practicing mindfulness for external (mindfully eating chocolate) and internal (observing thoughts) experiences, metaphors: “life on autopilot”, being a “distant observer”  Source: ACT for psychosis [28] |
| 7 | **How our mind works**  Objective: Developing a different relationship towards thoughts by understanding that they mostly consist of automatic rules and judgments learned in our past, giving thoughts less power dictating our behaviour  Target mechanism: Goal-orientated action planning | Debunking thoughts by distinguishing between facts and appraisals (Bad Cup), noticing automaticity and uncontrollability of thoughts (“Mary had a little lamb” and “Don’t think of a pink elephant”) and acting contrary to thoughts (“Don’t do what your mind says”), metaphors: mind as a production machinery and hard drive with data garbage  Source: ACT metaphors [29] and ACT for life [30] |
| 8 | **Helpful vs. unhelpful thoughts**  Objective: Distinguishing between helpful and unhelpful internal experiences and learning to act contrary to them without trying to avoid or control them  Target mechanism: Disidentification | Classifying everyday thoughts in unhelpful and helpful thoughts, actively executing defusion in “Taking your mind for a walk”, metaphors: thoughts as ankle cuffs vs. tools  Source: ACT for psychosis [28] |
| 9 | **Defusion techniques**  Objective: Learning to actively distance from internal experiences by using cognitive and behavioural strategies  Target mechanism: Self-regulation | Trying out different defusion and detached mindfulness techniques e.g. “labeling thoughts”, “floating leaves on a stream” and “Attention training technique” and choosing one for the “instruction manual for the mind”, metaphors: mind as parrot always telling the same story, the little “mind monster”  Source: ACT metaphors [29], ACT for psychosis [28], Metacognitive Therapy for anxiety and depression [31] |

*Note.* Reprinted from “Developing a mechanism-based therapy for acute psychiatric inpatients with psychotic symptoms: An Intervention Mapping approach” [7].

## Supplementary Table 2. Participants’ attendance rates and reasons for non-attendance

| Session | 1 | 2 | 3 | 4 | 5 | 6 | 7 | 8 | 9 |
| --- | --- | --- | --- | --- | --- | --- | --- | --- | --- |
| Attendance N (missing n) | 36 (2) | 37 (5) | 37 (1) | 37 (4) | 37 (5) | 35 (4) | 35 (5) | 35 (5) | 35 (8) |
| Attendance rate (%) | 94.4 | 86.5 | 97.3 | 89.2 | 86.5 | 88.6 | 85.7 | 85.7 | 77.1 |
| Reason for non-attendance, n |  |  |  |  |  |  |  |  |  |
| Lack of motivation | 1 | 1 |  |  | 1 | 1 | 1 | 1 | 1 |
| Non-capable |  | 1 | 1 | 1 | 2 |  | 1 | 2 | 4 |
| Isolation room | 1 |  |  |  |  |  |  |  |  |
| Other appointment |  | 3 |  | 2 | 2 | 2 | 2 | 2 | 2 |
| Disorganized |  |  |  | 1 |  | 1 | 1 |  | 1 |

*Note.* The N for attendance refers to all participants who hypothetically could have participated in the session, i.e., still participated in the study in the corresponding module (see Consort Flow Diagram). 36 of the 37 participants were in the study for Module I (Session 1), 37 for Module II (Session 2-6), and 35 for Module III (Session 6-9). The mean group therapy dose received by participants was 465.4 minutes (*SD* = 93.2), corresponding to eight sessions.

## Supplementary Table 3. Participants’ qualitative feedback on the feedback questionnaires of each module and the overall intervention

| Item | Psychoeducation | Cognitive Insight | Cognitive Defusion | Overall |
| --- | --- | --- | --- | --- |
| Feedback provided on insights from the modules, N (missing n) | 34 (7) | 37 (8) | 35 (11) | 35 (16) |
| Unspecific answers  (example quotations) | “The therapist was very caring.” (P20)  “This gave me motivation to fight.” (P56)  “More fun in life, skills for healing.”(P37) | “More inner calmness.” (P30)  “Strengthened self-worth.” (P70)  “The construct of every session.” (P64) | “Allow myself more rest, want to stop drinking that much coffee.” (P30)  “One should get help when having problems.” (P90) | “Very helpful for the future.” (P77)  “New experiences on with other people.” (P05)  “Nothing.” (P46)  “Interest and motivation.” (P89) |
| Specific answers  (example quotations) | “Thoughts influence behaviour.” (P61)  “I can change something about the way I think and therewith, I can change my problems.” (P80)  “Learning how to deal with thoughts is important.” (P44) | “I will focus more on myself.” (P26)  “Careful with JTC, wait until you know what the other wants.” (P53)  “My judgment is influenced by emotional factors. In order to not harm myself, I must not decide hastily and under pressure, but evaluate calmly. Keyword thinking traps.” (P66)  “Not to cling to thoughts and go into the thought trap.” (P77) | “One can learn to treat thoughts differently.” (P49)  “Notice my thoughts actively and distinguish whether they are helpful or not and how much they influence my behaviour.” (P33)  “I don’t have to control my thoughts, thoughts are thoughts and not facts.” (P58) | “I learned how to differentiate between helpful and not helpful thoughts.” (P96)  “I can steer my thoughts.” (P28)  “The group helped me to see that many fight against the same problems and that there are many ways to cope with them.” (P22)  “Taking metacognitive per-spective, balancing thoughts, not taking decisions with too few information.” (P47) |
| Feedback provided on missing topics in the modules, N (missing n) | 34 (26) | 37 (27) | 35 (12) | 35 (30) |
| Unspecific answers  (example quotations) | “The interior of my pockets.” (P28)  “There were too few participants.” (P39) | - | “Some things were too fast.” (P58) | “Personal topics and examples.” (P53)  “Talking about topics in individual session to recognize what helps me.” (P20) |
| Specific answers  (example quotations) | “Thoughts versus voices.” (P80)  “Skills and how to stop thoughts.” (P20) | “How to handle incomplete information, decision aids for accepting things.” (P24)  “I need more tips on how to train my memory. I know this doesn't fit with the problems of the others.” (P16)  “We only talked about thoughts, I would be interested also in auditory hallucinations, do we handle them just the same way as thoughts?” (P80) | “Discuss thoughts during acute psychosis.” (P49)  “How do I differentiate between helpful and not helpful if thoughts are very complex?” (P24) | “Social competencies.” (P08) |

## Supplementary Table 4. Participation in supplementary treatments additionally to the experimental group therapy

| Supplementary treatments (multiple therapies possible), n (%) | 37 (100) |
| --- | --- |
| Individual psychotherapy | 37 (100) |
| Other group therapy | 26 (72.9) |
| Occupational therapy | 37 (100) |
| Sports therapy | 28 (75.7) |
| Electroconvulsive therapy | 5 (13.5) |

*Note.* Participants were able to take part in one other group therapy module besides the experimental group intervention. Supplementary group therapies included a transdiagnostic Skillstraining, a Resource Group, a Psychoeducation Group and a Crisis-competence Group (see Supplementary Methods 1). All participants received individual sessions. The accumulated mean therapy dose including group therapy and individual sessions resulted in a total therapy dose of 711.3 minutes (SD = 103.55) during the study period.

## Supplementary Table 5. Content of individual psychotherapy sessions during the study period

| ID | Individual therapy dose (minutes) | Therapy goals | Topics and therapeutic techniques related to group | Topics and therapeutic techniques not related to group | Homework |
| --- | --- | --- | --- | --- | --- |
| P41 | 250 | 1. Tension regulation  2. Value exploration | 1. Defusion techniques  2. Value commitment | 1. Crisis formulation  2. Skillstraining | 1. Skillstraining |
| P89 | 300 | 1. Distancing to thoughts  2. Behavioural activation | 1. Identifying unhelpful thoughts  2. Defusion techniques | 1. Crisis formulation  2. Behavioural activation  3. Emotion regulation  4. Trauma exploration | 1. Defusion techniques |
| P53 | 400 | 1. Grief management  2. Value exploration | 1. Acceptance  2. Defusion techniques  3. Value commitment | 1. Crisis formulation  2. Psychoeducation depression  3. Behavioural activation | 1. Grief exposition  2. Defusion techniques  2. Positive activities |
| P26 | 250 | 1. Stress management  2. Distancing to thoughts | 1. Psychoeducation psychosis  2. Defusion techniques | 1. Crisis formulation  2. Behavioural activation | None |
| P49 | 250 | 1. Stress management | - | 1. Crisis formulation  2. Stress management  3. Relapse prevention | 1. Stress management |
| P33 | 300 | 1. Psychoeducation  2. Stress management | 1. Psychoeducation psychosis  2. Defusion techniques | 1. Crisis formulation  2. Relapse prevention | 1. Stress management |
| P64 | 250 | 1. Distancing to thoughts  2. Behavioural activation | 1. Acceptance  2. Defusion techniques | 1. Crisis formulation  2. Behavioural activation  3. Relaxation techniques | 1. Defusion techniques  2. Relaxation techniques |
| P03 | 300 | 1. Distancing to voices  2. Value exploration | 1. Thought disputation  2. Defusion techniques  3. Value commitment | 1. Crisis formulation  2. Motivational interviewing  3. Behavioural activation | 1. Defusion techniques |
| P77 | 200 | 1. Distancing to thoughts  2. Emotion regulation | 1. Defusion techniques | 1. Crisis formulation  2. Emotion regulation  3. Relaxation techniques | 1. Defusion techniques  2. Relaxation techniques |
| P37 | 150 | 1. Tension regulation | - | 1. Crisis formulation  2. Skillstraining  3. Relapse prevention | 1. Organization further treatment |
| P83 | 250 | 1. Distancing to thoughts  2. Emotion regulation | 1. Defusion techniques  2. Acceptance | 1. Crisis formulation  2. Skillstraining  3. Social competencies  4. Behavioural activation | 1. Defusion techniques  2. Skillstraining  3. Behavioural activation |
| P08 | 250 | 1. Distancing to thoughts  2. Emotion regulation | 1. Psychoeducation psychosis | 1. Crisis formulation  2. Stress management  3. Social competencies | 1. Relapse prevention |
| P58 | 200 | 1. Distancing to thoughts | 1. Psychoeducation psychosis  2. Defusion techniques | 1. Crisis formulation | 1. Defusion techniques |
| P24 | 200 | 1. Stress management | 1. Acceptance | 1. Crisis formulation  2. Emotion regulation  3. Trauma exploration | - |
| P96 | 200 | 1. Social competencies  2. Behavioural activation | 1. Defusion techniques  2. Acceptance | 1. Crisis formulation  2. Biographical work  3. Diagnostic clarification | 1. Behavioural activation |
| P30 | 200 | 1. Value clarification  2. Behavioural activation | 1. Defusion techniques  2. Value commitment | 1. Crisis formulation  2. Social competencies | 1. Problem solving techniques |
| P74 | 350 | 1. Tension regulation  2. Value clarification | 1. Defusion techniques  2. Acceptance  3. Value commitment | 1. Crisis formulation  2. Skillstraining  3. Behavioural activation | 1. Defusion techniques  2. Skillstraining |
| P61 | 250 | 1. Illness acceptance  2. Emotion regulation | 1. Thought disputation  2. Defusion techniques  3. Value commitment | 1. Crisis formulation  2. Emotional exposition | 1. Behavioural activation |
| P28 | 300 | 1. Psychoeducation  2. Sleeping hygiene  3. Distancing to thoughts | 1. Psychoeducation psychosis  2. Thought disputation  3. Defusion techniques | 1. Crisis formulation  2. Sleep hygiene | 1. Thought disputation  2. Defusion techniques |
| P80 | 250 | 1. Distancing to thoughts  2. Behavioural activation | 1. Thought disputation | 1. Crisis formulation  2. Motivational interviewing  3. Relapse prevention | 1. Relapse prevention |
| P06 | 250 | 1. Improving self-esteem | 1. Value exploration  2. Defusion techniques | 1. Crisis formulation  2. Diagnostic clarification | 1. Defusion techniques |
| P93 | 250 | 1. Distancing to thoughts  2. Emotion regulation | 1. Thought disputation  2. Defusion techniques | 1. Crisis formulation  2. Emotional exposition  3. Behavioural activation | 1. Defusion techniques  2. Behavioural activation |
| P46 | 250 | 1. Value commitment | 1. Psychoeducation psychosis | 1. Crisis formulation  2. Trauma exploration | - |
| P16 | 200 | 1. Value commitment | 1. Thought disputation | 1. Crisis formulation  2. Self care | - |
| P86 | 300 | 1. Distancing to hallucinations  2. Stress management | 1. Defusion techniques | 1. Crisis formulation  2. Biographical work | - |
| P05 | 200 | 1. Psychoeducation  2. Behavioural activation | 1. Psychoeducation psychosis | 1. Crisis formulation | - |
| P39 | 200 | 1. Behavioural activation  2. Grief management | - | 1. Crisis formulation  2. Grief exposition | 1. Grief work |
| P20 | 250 | 1. Distancing to thoughts | 1. Psychoeducation psychosis  2. Thought disputation | 1. Crisis formulation | 1. Thought disputation |
| P11 | 150 | 1. Distancing to hallucinations | 1. Defusion techniques | 1. Crisis formulation  2. Behavioural activation | 1. Defusion techniques |
| P90 | 250 | 1. Distancing to thoughts  2. Psychoeducation | 1. Psychoeducation psychosis | 1. Crisis formulation  2. Stress management  3. Resource activation | 1. Social competencies |
| P22 | 250 | 1. Distancing to thoughts  2. Psychoeducation | 1. Psychoeducation psychosis  2. Thought disputation | 1. Crisis formulation  2. Behavioural activation  2. Stress management | 1. Behavioural activation |
| P44 | 350 | 1. Illness acceptance  2. Behavioural activation | 1. Psychoeducation psychosis | 1. Crisis formulation  2. Social competencies  3. Emotion regulation  4. Behavioural activation | 1. Behavioural activation |
| P66 | 250 | 1. Distancing to thoughts | 1. Thought disputation  2. Defusion techniques | 1. Crisis formulation  2. Psychoeducation depression | 1. Defusion techniques  2. Behavioural activation |
| P47 | 250 | 1. Distancing to thoughts  2. Distancing to voices | 1. Defusion techniques | 1. Crisis formulation  2. Stress management | 1. Defusion techniques |
| P70 | 200 | 1. Psychoeducation  2. Distancing to thoughts | 1. Psychoeducation psychosis  2. Defusion techniques  3. Value clarification | 1. Crisis formulation  2. Relapse prevention | 1. Defusion techniques |
| P56 | 200 | 1. Psychoeducation  2. Distancing to thoughts | 1. Psychoeducation psychosis  2. Acceptance | 1. Crisis formulation | - |
| P02 | 200 | 1. Psychoeducation  2. Distancing to thoughts | 1. Thought disputation  2. Defusion techniques | 1. Social competencies | 1. Thought disputation |
|  |  |  |  |  |  |

## Supplementary Table 6. Transcript notes of individual quotes supporting the thematic analysis for each question in the semi-structured interview

| ID | Transcript notes of quotes | | | |
| --- | --- | --- | --- | --- |
|  | Like | Insights | Dislike | Study and group setup |
| P33 | Good examples, felt taken serious, practical exercises and theory, Taking your mind for a walk | Don't take every thought serious, Understanding how mind works | Nothing | Reduce group size, Offer group in the morning |
| P64 | Exciting topics, techniques how to deal with thoughts | Thoughts are not always helpful, letting the mind talk, letting thoughts pass, willingness | Sometimes too slow | Too many questionnaires |
| P03 | Interesting topics, defusion,  mindfulness | Setting priorities, take decisions according to values | Nothing | Everything fine |
| P77 | Clear structure, comprehensible, great group, great examples | Detecting thought patterns, treating myself different in the future, slowing down, being mindful, thinking first | Sometimes too fast | Everything fine |
| P37 | Good structure, reflecting on thoughts | Reflecting own thoughts, accepting negative thoughts, focusing on positive thoughts, noticing thoughts | Sometimes too slow | Reduce group size |
| P08 | Very good group | Knowing what thoughts are, meta-perspective, staying focused, stopping autopilot | Lack of commitment from some participants | Everything fine |
| P58 | Helpful techniques | Unhelpful vs. helpful thoughts, fact vs. appraisal | Complicated terms, too fast, too many topics | Everything fine |
| P24 | Clear structure, exchange with patients, motivation through therapist, pictures and examples | Defusion techniques | Topics treated too superficial, more time needed, more information on illness needed, more examples on psychosis | Some questionnaires redundant |
| P96 | Good structure, different modules | Looking at things from a different ankle, unhelpful vs. helpful thoughts | Breaks in the middle needed | Everything fine |
| P30 | Felt comfortable in group, defusion | Don’t remember anything | Nothing | Was ok |
| P61 | Interesting topics, felt comfortable in group, new ways of dealing with thoughts, feeling enthusiastic | Mindfulness, defusion techniques, imagination exercises | Nothing | Everything fine |
| P28 | Information supply, looking down on thoughts | Butterfly exercise, distancing from thoughts | Nothing | Everything fine |
| P06 | Clear structure | Don’t take thoughts so serious, focusing on important goals, defusion techniques | Sometimes too slow, topics too easy | Everything fine |
| P46 | Interesting topics, clear structure, good offers, good treatment | Forgot things right away | Nothing | Everything fine |
| P16 | Good examples, great therapist, other patients in group, structure of the group | Don't take every thought serious | Sometimes too slow, more examples and exercises needed | Everything fine |
| P86 | Group in general, good topics, good exercises | Problems remembering stuff | More time needed, more practical exercises needed | Everything fine |
| P05 | Good topics, exchange with others, talking openly | Nice memories | Nothing | Everything fine |
| P39 | General satisfaction with group | Learning to rethink | Lack of commitment from some participants | Everything fine |
| P20 | Practical exercises, exchange with others | Learning new coping mechanisms, using defusion techniques | Too much theory, more exercises needed | Questionnaires hard to answer |
| P22 | Sharing with others, coping strategies | Talking about problems earlier, defusion techniques, monster metaphor, asking for help right away | Sometimes hard to open up | Questionnaires hard to answer |
| P66 | Empathic therapist, sharing with others, simple exercises, great group | Recognizing thinking patterns, distancing from thoughts, directing attention, butterfly metaphor | More time for sessions needed, more focus on therapy projects | Everything fine |
| P47 | Mindfulness, talking openly, exchange in the group | Therapy cards, act vs. appraisals Collecting enough information before taking a decisions | Sometimes too long | Everything fine |
| P70 | Exchange with others, recognizing variety of viewpoints | Defusion techniques, butterfly metaphor | Nothing | Group should be longer than 60 minutes, more groups per week |
| P02 | Liked all topics | Recognizing emotions in others, defusion techniques, mindfulness | Group time too late | Shorter sessions |

## Supplementary Table 7. Codes identified for each participant for the thematic analysis of the semi-structured interview

| ID | Codes Like | Codes Insights | Codes Dislike | Codes Setup |
| --- | --- | --- | --- | --- |
| P33 | 1. Good examples 4. Felt taken serious 2. Practical exercises and theory 3. Walking your mind | 1. Don't take every thought serious 2. Understanding how mind works | Does not apply | 1. Reduce group size 2. Offer group in the morning |
| P64 | 1. Exciting  2. Techniques how to deal with thoughts | 1. Thoughts are not always helpful 2. Letting the mind talk 3. Letting thoughts pass 4. Willingness | 1. Sometimes to slow | 1. Too many questionnaires |
| P03 | 1. Interesting topic 2. Defusion  3. Mindfulness | 1. Setting priorities 2. Make decisions according to values | Does not apply | Does not apply |
| P77 | 1. Clear structure  2. Comprehensible  3. Great group 4. Great examples | 1. Detecting thought patterns 2. Treating myself different in the future 3. Slowing down  4. Being mindful  5. Think first, act then | 1. Sometimes too fast | Does not apply |
| P37 | 1. Good structure 2. Reflecting thoughts | 1. Reflect thoughts 2. Accept negative thoughts 3. Focus on positive thoughts 4. Notice thoughts | 1. Sometimes to slow | 1. Reduce group size |
| P08 | 1. Very good group | 1. Knowing what thoughts are 2. Metaperspective 3. Stay focuses 4. Stop autopilot | 1. Lack of commitment from participants | Does not apply |
| P58 | 1. Helpful techniques | 1. Unhelpful vs. helpful thoughts 2. Fact vs. appraisal | 1. Complicated terms  2. Too fast  3. Too many topics | Does not apply |
| P24 | 1. Clear structure 2. Exchange with patients 3. Motivation through therapist 4. Pictures and examples | 1. Defusion techniques | 1. Topics to superficial 2. More time needed 3. More information on illness needed 4. More examples on psychosis | 1. Some questionnaires redundant |
| P96 | 1. Good structure  2. Different modules | 1. Look at things from a different ankle 2. Unhelpful vs. helpful thoughts | 1. Breaks in the middle needed | Does not apply |
| P30 | 1. Comfortable in group 2. Defusion | Does not apply | Does not apply | Does not apply |
| P61 | 1. Interesting  2. Comfortable in group 3. New ways of dealing with thoughts 4. Feeling enthusiastic | 1. Mindfulness  2. Defusion techniques  3. Imaginations | Does not apply | Does not apply |
| P28 | 1. Information  2. Looking down on thoughts | 1. Butterfly exercise  2. Distance to thoughts | Does not apply | Does not apply |
| P06 | 1. Clear structure | 1. Don’t take thoughts so serious 2. Focus on important goals  3. Defusion techniques | 1. Sometimes too slow  2. Too easy | Does not apply |
| P46 | 1. Interesting topics 2. Clear structure  3. Good offers 4. Good treatment | 1. Forgot things right away | Does not apply | Does not apply |
| P16 | 1. Good examples  2. Great therapist  3. Patients in group 4. Structure of the group | 1. Don't take every thought serious | 1. Sometimes to slow 2. More examples and exercises | Does not apply |
| P86 | 1. Group 2. Good topics 3. Good exercises | 1. Problems remembering stuff | 1. More time needed  2. More practical exercises | Does not apply |
| P05 | 1. Good topics 3. Exchange with others  2. Talk openly | 1. Nice memories | Does not apply | Does not apply |
| P39 | 1. General satisfaction | 1. Learning to rethink | 1. Lack of commitment from participants | Does not apply |
| P20 | 1. Practical exercises  2. Exchange with others | 1. Coping mechanisms 2. Using inner assistant | 1. Too much theory 2. More exercises needed | 1. Questionnaires hard to answer |
| P11 | Does not apply | Does not apply | Does not apply | Does not apply |
| P22 | 1. Sharing with others  2. Coping strategies | 1. Talk about problems 2. Defusion techniques 3. Monster metaphor 4. Consulting help right away | 1. Sometimes had to open up | 1. Questionnaires hard to answer |
| P66 | 1. Empathic therapist 2. Sharing with others 3. Discreet 4. Great group | 1. Recognizing patterns 2. Distancing from thoughts 3. Directing attention 4. Butterfly metaphor | 1. More time for sessions 2. More focus in therapy projects | Does not apply |
| P47 | 1. Mindfulness 2. Talking openly  Exchange in the group | 1. Therapy cards 2. Fact vs. appraisals 3. Enough information for decisions | 1. Sometimes too long | Does not apply |
| P70 | 1. Exchange with others 2. Recognizing variety of viewpoints | 1. Defusion techniques 2. Butterfly metaphor | Does not apply | 1. Group should be longer than 60 min 2. More groups per week |
| P02 | 1. Liked topics | 1. Recognizing emotions in others 2. Defusion techniques 3. Mindfulness | 1. Group time too late | 1. Shorter sessions |

## Supplementary Table 8. Recommendations for future research

| **Observation** | **Recommended change(s)** |
| --- | --- |
| **Recruitment and retention**   - Eligibility rate: 75.8% - Consent rate: 78.7% - Trial entry rate: 100% - Completion rate: 99.4% - Retention rate: 89.2%   Participants were only recruited from one ward | Increase the eligibility rate by screening all admitted patients with PSDs. Involve entire clinician team in screening. Expand study to participants from other acute psychiatric inpatient wards to ensure adequate pool of participants  Include different wards in the same hospital and different recruitment sites across Germany |
| **Eligibility criteria**  Missing eligibility for psychotherapy was not clearly defined making the inclusion/exclusion decisions after screening challenging | Clearly operationalize eligibility for psychotherapy (e.g. scores on the PANSS ≥ 5 in hostility and uncooperative and ≥6 in suspiciousness) [32] in a future RCT still keeping in mind the acute setting and research question |
| **Outcome measures**  The WHODAS turned out to be unsuitable as a sole self-report measure for inpatients with acute psychosis, as they tended to over-estimate their functioning at baseline [33, 34]. No significant correlations were found between the self-report and rater-adjusted WHODAS-2.0 scores and GAF at baseline (r = 0.056, 95% CI = -0.27, 0.37, p = 0.739; r = -0.26, 95% CI = -0.54, 0.07, p = 0.115). However, both self-report and rater-adjusted WHODAS-2.0 scores and GAF post-intervention were significantly correlated with a much larger correlation associated with rater-adjustment (r = -0.34, 95% CI = -0.60, -0.01, p = 0.042; r = -0.67, 95% CI = -0.82, -0.45, p < 0.001)  The CGI was too unspecific to measure treatment success given the specific psychopathology (positive and negative symptoms) of the target group [35]  Outcome measures mostly focused on symptom change with few focusing on the overarching treatment goals of recovery or crisis reduction [36]  Psychological mechanisms were only measured by two process outcomes (BCIS and CFQ), making it difficult to make sophisticated statements about the hypothesized therapeutic mechanisms  Modules’ feedback questionnaires were hard to answer for same participants and some questions were found to be redundant  Outcome measures were rated by clinicians involved in the overall treatment of the patients | Identify additional quality of life assessments such as the Recovering Quality of life that are more suitable for the target group [37]  Use the CGI-Schizophrenia Scale in order to asses more specific treatment effects [38]  Include recovery oriented outcome measures like the Beck Hopelessness Scale or the Process of Recovery Questionnaire [39], experiences of crisis in psychosis [36] or Self-stigma of mental illness scale [11]  Add additional mechanism measures e.g. the Cognitive bias questionnaire for psychosis [40] and the Acceptance and Action questionnaire [41] to distinguish between correlated and overlapping treatment processes. Also add direct measures of cognitive biases e.g. using the BADE procedure to measure the jumping to conclusion (JTC) bias [42]  Shorten and simplify feedback questionnaires on modules, eliminate redundant questions  Make sure assessments are completed by research assistants blind to treatment allocation or not involved in the patient’s treatment |
| **Assessment time points**  Baseline diagnostic measures e.g. PANSS were only taken at the beginning and end of the treatment, no progression diagnostic in between took place  Mechanism measures were taken immediately after the respective module with no time for patients to progress or practice therapy contents  No follow up measures were taken | Make sure to continuously assess outcome measures in order to map treatment effects over the entire treatment period  Make sure to assess process measures weekly but also in various follow up assessment  Establish follow-up measurements e.g. 6, 12, and 24 months month after treatment completion to account for long-term effects, and also collect data on readmissions to other hospitals |
| **External delivery framework**  Overall retention rate was high at 89.2% for all three modules, but shortening the intervention's duration would allow everyone to participate in all the content.  According to participants, therapy sessions took too long with 60 minutes and contained too much theoretical information  According to participants, sessions contained too many examples and exercises for the given time  Depending on the group composition, disturbances and unrest occurred that could not be solved by one therapist alone  Mostly clinical psychologist trained in CBT conducted the group therapy  According to participants, group sizes were sometimes too large. The average number of patients attending a session was 6, but due to the naturalistic setting of the study, 20 sessions out of 81 sessions over the entire study period were held with more than 6 patients.  According to participants, the meaning of group-specific terms like metacognition, fusion and defusion was hard to understand  Therapy contents were partly too theoretical with participants benefitting most from exercises and practical examples  Complementary individual sessions were not always coordinated with group contents  There was no restrictions for participants to take part in complementary psychosocial treatments | Shorten total amount of sessions to five with one psychoeducative session and two sessions in Module II and Module III  Shorten contents down to the basics and reduce therapy lengths to a maximum of 40 minutes. Make sure to allow for time buffer  Shorten number of examples and practical exercises down to the most helpful (according to participants’ feedback)  If possible, let two therapists conduct the therapy sessions  Train co-therapists from related professions such as occupational therapy or nursing. Properly manualise the group concept  Limit group size to a maximum of seven patients as suggested in the literature [27]. High therapy demand could for example be met by a second parallel group  Simplify therapy language and avoid using specialized terms by e.g. replacing metacognition with “thought distance” or “thinking about thinking” and defusion with “detachment”  Shorten theoretical input to a minimum and focus on practical exercises. Make sure examples fit the current crisis situation and are transferable into patients’ everyday life  Create a consistent approach for one-on-one sessions to make them comparable between participants  For the study period, limit participation to the group intervention and individual sessions |
| **Therapy contents**  Disorder related language was rarely used. Nevertheless, participants frequently asked about the role of psychotic symptoms and found it helpful to receive information  Module “Cognitive Insight” with four sessions was found to be lengthy with overlapping topics and examples (e.g. attribution styles and jumping to conclusions). Patients struggled to transfer given examples to their own psychotic experiences  Module “Cognitive Defusion” with four sessions was found to include too much theory compared to the amount of practical exercises. Participants reported difficulty in grasping the metacognitive concept, which treats delusions and hallucinations on par with "normal thoughts”  Therapy contents and therapeutic attitude clearly differed from the primary medical treatment focus of acute psychiatric inpatient wards making a unified treatment approach difficult. As a result, patients partly received contradictory information on treatment goals e.g. symptom reduction vs. symptom acceptance | Include more psychoeducative information about symptoms of psychosis and give room for discussion and exchange  Shorten amount and content of sessions e.g. to two sessions and include only examples found most helpful for participants. Make sure to include relevant psychosis-related examples next to “neutral” ones and encourage sharing personal experiences  Shorten theoretical input to a minimum and focus on practicing and revising exercises with patients’ own examples. Make sure to give enough psychoeducative information about the classification of psychotic experiences from a metacognitive philosophy  Provide trainings for the whole treatment team to integrate psychological thinking, formulation and hypothesizing in the treatment plan. Make sure to educate the whole team about the treatment model behind the group intervention and frequently exchange in interdisciplinary team meetings [43] |
| **Treatment fidelity**  Sessions followed a manual, but were not audio recorded to ensure therapist’s adherence to the treatment model  **Health economics**  The costs of training, intervention delivery and analysis were integrated into routine clinical care and only sufficient to conduct a feasibility study  **Statistical analysis**  Given the small sample size, statistical analysis only included pre-post evaluations to test for preliminary effectiveness. No mediation analysis was included to test the effect of mechanisms of change | Audio record group and individual sessions to ensure treatment fidelity  Calculate costs relevant for a randomized controlled pilot study and a subsequent fully powered trial involving multiple sites. Aim to build up research cooperations and raise research funds  In a larger scale study, include analysis of moderators and mediators to account for change mechanism effects |
| **Study design for a future research**  The study design was uncontrolled  Assessments were non-blinded  Sample size was small | Set up control arm to test the specificity of change mechanisms in the experimental group intervention [44–46]  Make sure raters and therapists are different researches and ensure raters are blinded to treatment allocation  Ensure pilot trial sample size is sufficiently large to achieve medium effect sizes e.g. with at least 15 participants per treatment arm [47] |

# References

1. Wood L, Williams C, Billings J, Johnson S (2019) The therapeutic needs of psychiatric in-patients with psychosis: A qualitative exploration of patient and staff perspectives. BJPsych Open 5:e45. https://doi.org/10.1192/bjo.2019.33

2. Griffiths R, Mansell W, Edge D, Tai S (2019) Sources of Distress in First-Episode Psychosis: A Systematic Review and Qualitative Metasynthesis. Qual Health Res 29:107–123. https://doi.org/10.1177/1049732318790544

3. Bowers L, Chaplin R, Quirk A, Lelliott P (2009) A conceptual model of the aims and functions of acute inpatient psychiatry. J Ment Health 18:316–325. https://doi.org/10.1080/09638230802053359

4. Barnicot K, Michael C, Trione E, et al (2020) Psychological interventions for acute psychiatric inpatients with schizophrenia-spectrum disorders: A systematic review and meta-analysis. Clin Psychol Rev 82:101929. https://doi.org/10.1016/j.cpr.2020.101929

5. Bartholomew Eldredge LK (2016) Planning health promotion programs: an intervention mapping approach, Fourth edition. Jossey-Bass & Pfeiffer Imprints, Wiley, San Francisco, CA

6. Teresi JA, Yu X, Stewart AL, Hays RD (2022) Guidelines for Designing and Evaluating Feasibility Pilot Studies. Med Care 60:95–103. https://doi.org/10.1097/MLR.0000000000001664

7. Gussmann E, Lucae S, Falkai P, et al (2023) Developing a mechanism-based therapy for acute psychiatric inpatients with psychotic symptoms: an Intervention Mapping approach. Frontiers in Psychiatry 14. <https://doi.org/10.3389/fpsyt.2023.1160075>

8. Bleijenberg N, de Man-van Ginkel JM, Trappenburg JCA, et al (2018) Increasing value and reducing waste by optimizing the development of complex interventions: Enriching the development phase of the Medical Research Council (MRC) Framework. Int J Nurs Stud 79:86–93. https://doi.org/10.1016/j.ijnurstu.2017.12.001

9. El-Hagrassy MM, Duarte D, Thibaut A, et al (2018) Principles of Designing a Clinical Trial: Optimizing Chances of Trial Success. Curr Behav Neurosci Rep 5:143–152. https://doi.org/10.1007/s40473-018-0152-y

10. Kazdin AE (2007) Mediators and Mechanisms of Change in Psychotherapy Research. Annu Rev Clin Psychol 3:1–27. https://doi.org/10.1146/annurev.clinpsy.3.022806.091432

11. Corrigan PW, Michaels PJ, Vega E, et al (2012) Self-stigma of mental illness scale—short form: Reliability and validity. Psychiatry Res 199:65–69. https://doi.org/10.1016/j.psychres.2012.04.009

12. Hofmann SG, Hayes SC (2019) The Future of Intervention Science: Process-Based Therapy. Clin Psychol Sci 7:37–50. https://doi.org/10.1177/2167702618772296

13. Herpertz S, Schramm E, Deisenhofer A-K (2022) Modulare Psychotherapie: ein Mechanismus-basiertes, personalisiertes Vorgehen. Schattauer, Stuttgart

14. Bäuml J (2016) Handbuch der Psychoedukation: für Psychiatrie, Psychotherapie und Psychosomatische Medizin; mit ... 61 Tabellen. Schattauer, Stuttgart

15. Bohus M, Wolf-Arehult M (2016) Interaktives Skillstraining für Borderline-Patienten: das Therapeutenmanual: mit 158 Info- und Arbeitsblättern, 2., aktualisierte und erweiterte Auflage. Schattauer, Stuttgart

16. Hofheinz C, Heidenreich T, Michalak J (2017) Werteorientierte Verhaltensaktivierung bei depressiven Störungen: Therapiemanual: mit E-Book inside und Arbeitsmaterial, 1. Auflage. Beltz, Weinheim Basel

17. Lindenmeyer J (2021) Alkoholabhängigkeit, Angststörungen, Arbeitstherapie, Genusstraining, Männliche Sexualität und Partnerschaft, Nachsorge, Partnerseminar, Pathologischer PC- und Internetgebrauch, Pathologisches Glücksspiel, Raucherentwöhnung, Stress am Arbeitsplatz: mit E-Book inside und Arbeitsmaterial, 3., überarbeitete und erweiterte Auflage. Beltz, Weinheim Basel

18. Ebrahimi A, Poursharifi H, Dolatshahi B, et al (2021) The Cognitive Model of Negative Symptoms in Schizophrenia: A Hierarchical Component Model With PLS-SEM. Front Psychiatry 12:707291. https://doi.org/10.3389/fpsyt.2021.707291

19. Freeman D, Garety PA, Kuipers E, et al (2002) A cognitive model of persecutory delusions. Br J Clin Psychol 41:331–347. https://doi.org/10.1348/014466502760387461

20. Garety PA, Kuipers E, Fowler D, et al (2001) A cognitive model of the positive symptoms of psychosis. Psychol Med 31:189–195. https://doi.org/10.1017/S0033291701003312

21. Kircher TTJ, Koch K, Stottmeister F, Durst V (2007) Metacognition and Reflexivity in Patients with Schizophrenia. Psychopathology 40:254–260. https://doi.org/10.1159/000101730

22. Alegría M, NeMoyer A, Falgàs Bagué I, et al (2018) Social Determinants of Mental Health: Where We Are and Where We Need to Go. Curr Psychiatry Rep 20:95. https://doi.org/10.1007/s11920-018-0969-9

23. Larøi F, Woodward TS (2007) Hallucinations from a Cognitive Perspective. Harv Rev Psychiatry 15:109–117. https://doi.org/10.1080/10673220701401993

24. Bach PA, Gaudiano B, Pankey J, et al (2006) Acceptance, mindfulness, values, and psychosis: applying acceptance and commitment therapy (ACT) to the chronically mentally ill. In: Mindfulness-Based Treatment Approaches. Elsevier, pp 93–116

25. Jelinek L, Hauschildt M, Moritz S (2015) Metakognitives Training bei Depression (D-MKT): mit E-Book inside und Trainingsmaterial, 1. Aufl. Beltz, Weinheim

26. Moritz S, Woodward TS (2007) Metacognitive training in schizophrenia: from basic research to knowledge translation and intervention: Curr Opin Psychiatry 20:619–625. https://doi.org/10.1097/YCO.0b013e3282f0b8ed

27. Fischer R, Scheunemann J, Bohlender A, et al (2022) ‘You are trying to teach us to think more slowly!’: Adapting Metacognitive Training for the acute care setting—A case report. Clin Psychol Psychother cpp.2755. https://doi.org/10.1002/cpp.2755

28. Pearson A, Tingey R (2011) ACT for psychosis: A treatment protocol for group therapy. Parma, Italy

29. Stoddard JA, Afari N (2014) The big book of ACT metaphors: a practitioner’s guide to experiential exercises and metaphors in acceptance and commitment therapy. New Harbinger Publications, Oakland, CA

30. Johns LC, Oliver JE, Khondoker M, et al (2016) The feasibility and acceptability of a brief Acceptance and Commitment Therapy (ACT) group intervention for people with psychosis: The ‘ACT for life’ study. J Behav Ther Exp Psychiatry 50:257–263. https://doi.org/10.1016/j.jbtep.2015.10.001

31. Wells A (2011) Metacognitive therapy for anxiety and depression. Guilford, New York, NY

32. Birulés I, López-Carrilero R, Cuadras D, et al (2020) Cognitive Insight in First-Episode Psychosis: Changes during Metacognitive Training. J Pers Med 10:253. https://doi.org/10.3390/jpm10040253

33. Sabbag S, Twamley EW, Vella L, et al (2012) Predictors of the accuracy of self assessment of everyday functioning in people with schizophrenia. Schizophr Res 137:190–195. https://doi.org/10.1016/j.schres.2012.02.002

34. Gspandl S, Peirson RP, Nahhas RW, et al (2018) Comparing Global Assessment of Functioning (GAF) and World Health Organization Disability Assessment Schedule (WHODAS) 2.0 in schizophrenia. Psychiatry Res 259:251–253. https://doi.org/10.1016/j.psychres.2017.10.033

35. Leucht S, Davis JM, Engel RR, et al (2009) Definitions of response and remission in schizophrenia: recommendations for their use and their presentation. Acta Psychiatr Scand 119:7–14. https://doi.org/10.1111/j.1600-0447.2008.01308.x

36. Wood L, Williams C, Pinfold V, et al (2022) Crisis-focused Cognitive Behavioural Therapy for psychosis (CBTp) in acute mental health inpatient settings (the CRISIS study): protocol for a pilot randomised controlled trial. Pilot Feasibility Stud 8:205. https://doi.org/10.1186/s40814-022-01160-7

37. Keetharuth AD, Brazier J, Connell J, et al (2018) Recovering Quality of Life (ReQoL): a new generic self-reported outcome measure for use with people experiencing mental health difficulties. Br J Psychiatry 212:42–49. https://doi.org/10.1192/bjp.2017.10

38. Haro JM, Kamath SA, Ochoa S, et al (2003) The Clinical Global Impression-Schizophrenia scale: a simple instrument to measure the diversity of symptoms present in schizophrenia: CGI-SCH validity in the SOHO study. Acta Psychiatr Scand 107:16–23. https://doi.org/10.1034/j.1600-0447.107.s416.5.x

39. Beck AT, Weissman A, Lester D, Trexler L (1974) The measurement of pessimism: The Hopelessness Scale. J Consult Clin Psychol 42:861–865. https://doi.org/10.1037/h0037562

40. Peters ER, Moritz S, Schwannauer M, et al (2014) Cognitive Biases Questionnaire for Psychosis. Schizophr Bull 40:300–313. https://doi.org/10.1093/schbul/sbs199

41. Bond FW, Hayes SC, Baer RA, et al (2011) Preliminary Psychometric Properties of the Acceptance and Action Questionnaire–II: A Revised Measure of Psychological Inflexibility and Experiential Avoidance. Behav Ther 42:676–688. https://doi.org/10.1016/j.beth.2011.03.007

42. Moritz S, Woodward TS (2006) A generalized bias against disconfirmatory evidence in schizophrenia. Psychiatry Res 142:157–165. https://doi.org/10.1016/j.psychres.2005.08.016

43. Wood L, Williams C, Billings J, Johnson S (2019) The role of psychology in a multidisciplinary psychiatric inpatient setting: Perspective from the multidisciplinary team. Psychol Psychother Theory Res Pract 92:554–564. https://doi.org/10.1111/papt.12199

44. Garety P, Waller H, Emsley R, et al (2015) Cognitive Mechanisms of Change in Delusions: An Experimental Investigation Targeting Reasoning to Effect Change in Paranoia. Schizophr Bull 41:400–410. https://doi.org/10.1093/schbul/sbu103

45. Hoffart A, Johnson SU, Nordahl HM, Wells A (2018) Mechanisms of change in metacognitive and cognitive behavioral therapy for treatment-resistant anxiety: The role of metacognitive beliefs and coping strategies. J Exp Psychopathol 9:204380871878741. https://doi.org/10.1177/2043808718787414

46. Schlier B, Ludwig L, Wiesjahn M, et al (2020) Fostering coping as a mechanism of symptom change in cognitive behavioural therapy for psychosis. Schizophr Res 215:416–423. https://doi.org/10.1016/j.schres.2019.07.047

47. Whitehead AL, Julious SA, Cooper CL, Campbell MJ (2016) Estimating the sample size for a pilot randomised trial to minimise the overall trial sample size for the external pilot and main trial for a continuous outcome variable. Stat Methods Med Res 25:1057–1073. https://doi.org/10.1177/0962280215588241
